# Biological signatures and prediction of an immunosuppressive status—persistent critical illness—among orthopedic trauma patients using machine learning techniques

**DOI:** 10.3389/fimmu.2022.979877

**Published:** 2022-10-17

**Authors:** Mingxing Lei, Zhencan Han, Shengjie Wang, Chunxue Guo, Xianlong Zhang, Ya Song, Feng Lin, Tianlong Huang

**Affiliations:** ^1^ Department of Orthopedic Surgery, Hainan Hospital of Chinese People's Liberation Army (PLA) General Hospital, Sanya, China; ^2^ Chinese People's Liberation Army (PLA) Medical School, Beijing, China; ^3^ Department of Orthopedic Surgery National Clinical Research Center for Orthopedics, Sports Medicine and Rehabilitation, Chinese People's Liberation Army (PLA) General Hospital, Beijing, China; ^4^ Xiangya School of Medicine, Central South University, Changsha, China; ^5^ Department of Orthopedic Surgery, Shanghai Sixth People’s Hospital Affiliated to Shanghai Jiao Tong University, Shanghai, China; ^6^ Department of Biostatistics, Hengpu Yinuo (Beijing) Technology Co., Ltd, Beijing, China; ^7^ Department of Orthopedic, Xiangya Hospital of Central South University, Changsha, China; ^8^ Department of Orthopedic Surgery, The Second Xiangya Hospital of Central South University, Changsha, China

**Keywords:** machine learning, persistent critical illness, biological signature, lymphocytes, immunosuppression

## Abstract

**Background:**

Persistent critical illness (PerCI) is an immunosuppressive status. The underlying pathophysiology driving PerCI remains incompletely understood. The objectives of the study were to identify the biological signature of PerCI development, and to construct a reliable prediction model for patients who had suffered orthopedic trauma using machine learning techniques.

**Methods:**

This study enrolled 1257 patients from the Medical Information Mart for Intensive Care III (MIMIC-III) database. Lymphocytes were tracked from ICU admission to more than 20 days following admission to examine the dynamic changes over time. Over 40 possible variables were gathered for investigation. Patients were split 80:20 at random into a training cohort (n=1035) and an internal validation cohort (n=222). Four machine learning algorithms, including random forest, gradient boosting machine, decision tree, and support vector machine, and a logistic regression technique were utilized to train and optimize models using data from the training cohort. Patients in the internal validation cohort were used to validate models, and the optimal one was chosen. Patients from two large teaching hospitals were used for external validation (n=113). The key metrics that used to assess the prediction performance of models mainly included discrimination, calibration, and clinical usefulness. To encourage clinical application based on the optimal machine learning-based model, a web-based calculator was developed.

**Results:**

16.0% (201/1257) of all patients had PerCI in the MIMIC-III database. The means of lymphocytes (%) were consistently below the normal reference range across the time among PerCI patients (around 10.0%), whereas in patients without PerCI, the number of lymphocytes continued to increase and began to be in normal range on day 10 following ICU admission. Subgroup analysis demonstrated that patients with PerCI were in a more serious health condition at admission since those patients had worse nutritional status, more electrolyte imbalance and infection-related comorbidities, and more severe illness scores. Eight variables, including albumin, serum calcium, red cell volume distributing width (RDW), blood pH, heart rate, respiratory failure, pneumonia, and the Sepsis-related Organ Failure Assessment (SOFA) score, were significantly associated with PerCI, according to the least absolute shrinkage and selection operator (LASSO) logistic regression model combined with the 10-fold cross-validation. These variables were all included in the modelling. In comparison to other algorithms, the random forest had the optimal prediction ability with the highest area under receiver operating characteristic (AUROC) (0.823, 95% CI: 0.757-0.889), highest Youden index (1.571), and lowest Brier score (0.107). The AUROC in the external validation cohort was also up to 0.800 (95% CI: 0.688-0.912). Based on the risk stratification system, patients in the high-risk group had a 10.0-time greater chance of developing PerCI than those in the low-risk group. A web-based calculator was available at https://starxueshu-perci-prediction-main-9k8eof.streamlitapp.com/.

**Conclusions:**

Patients with PerCI typically remain in an immunosuppressive status, but those without PerCI gradually regain normal immunity. The dynamic changes of lymphocytes can be a reliable biomarker for PerCI. This work developed a reliable model that may be helpful in improving early diagnosis and targeted intervention of PerCI. Beneficial interventions, such as improving nutritional status and immunity, maintaining electrolyte and acid-base balance, curbing infection, and promoting respiratory recovery, are early warranted to prevent the onset of PerCI, especially among patients in the high-risk group and those with a continuously low level of lymphocytes.

## Introduction

The mortality of severe trauma patients has decreased due to advancements in trauma management and critical care (1). Patients who have survived the primary critical illness at the admission into intensive care unit (ICU) tended to develop a cascade of new complications that could lead to a secondary peak of “late deaths” (2–4). This trend was increasingly evident with improved survivorship due to advanced life support settings (2, 5). This transition into the state of chronic critical illness for ICU patients has yielded poor and prolonged recovery along with considerable expenditure of medical resources (6, 7), posing a new challenge for the critical care community.

Recently, the term “persistent critical illness (PerCI)” has been brought up to specify this phenotype of patients with chronic critical illness (8–11). The definition was based on the point where patient’s in-hospital mortality was more related to present health conditions rather than the severity of critical illness at admission (6, 12). Onset of PerCI occurred 10 days after ICU admission at a large population level (8, 11), which provided rational time-based epidemiological criteria for PerCI. The prevalence of PerCI was 5.0% to 33.9% in different ICU populations (8, 13, 14) and it was about 20% in severe trauma patients (15). Patients with this chronic condition were subject to a constellation of new clinical problems and suffer a propensity for late organ failures (9, 16). Consequently, compared with typical ICU patients, those patients had a greater mortality and a lower likelihood of being discharged from the ICU. Meanwhile, they required delicate critical care and consume vast health resources (8).

PerCI patients were characterized by immunosuppression (3), persistent inflammation (17), and endocrine dysfunction (18), all of which has been proposed as a mechanistic framework to explain the increase of PerCI in ICU settings. Persistent inflammation is often reflected by chronic low-grade inflammation, such as elevated interleukin-6 (IL-6) in the blood, immunosuppression mainly includes lymphocyte dysfunction and reduced antigen presentation, and catabolism contains defects in the metabolism of carbohydrate, lipid, and protein (19, 20). Recent studies revealed myeloid-derived suppressor cells, which had the power to affect nearly all host innate and adaptive immunity, were one of the key drivers of this persistent inflammation and immunosuppression (3). In addition, hematopoietic stem cells and myeloid-derived suppressor cells together in a vicious cycle of immune disorders to promote PerCI, especially in individuals with certain chronic conditions or aging (19).

Nonetheless, the underlying pathophysiology driving PerCI is still not fully understood. Early interventions might be particularly beneficial if potential candidates for PerCI could be identified in advance (21). Therefore, studies examining possible associations between biological signatures and the prevalence of PerCI were highly required. Data were extremely limited at the time, however, and a recent study pointed out that elevated urea-to-creatinine ratio might serve as a biochemical indicator of PerCI after acute major trauma (13). Additionally, IL-10 and interferon gamma-induced protein (IP-10) on day four could only minimally enhance PerCI prediction in patients with postoperative sepsis (22). Thus, without taking more variables into account, the biochemical signatures had limited application in identifying patients subjected to PerCI. Compared with sepsis patients, ICU trauma patients should be noted as a special population, since those patients were often younger and had less comorbid diseases, where phenotypes of prolonged length of ICU stay and PerCI might be more easily investigated and significantly distinct from factors relating to baseline frailty (21).

Therefore, this study sought to examine the biological signature of developing PerCI, particularly the dynamic changes of lymphocytes, and further to construct a reliable prediction model for PerCI using machine learning techniques. This study hypothesized that features of the change of lymphocytes over time after admission could be revealed in this study, some important biochemical contributors reflecting PerCI could be identified, and an accurate model with favorable prediction performance could be proposed based on risk factors.

## Methods

### Study design, inclusive and exclusive criteria

This study retrospective analyzed subjects in the Medical Information Mart for Intensive Care III (MIMIC-III) database (Version 1.4, https://physionet.org/content/mimiciii/1.4/). The MIMIC-III database includes identified health-related information of patients who were admitted into critical care units of the Beth Israel Deaconess Medical Center (23). The database is currently accessible through Google Cloud Platform and Amazon Web Services. Authors who complied with the requirements for access to the MIMIC-III database were approved by PhysioNet [20]. In the study, the author completed all training courses including the Human Research, Data or Specimens Only Research, and 1-Basic Course, and acquired the data user requirements (Record ID: 32128436). Users can access to the data on the cloud by simply adding the appropriate cloud identifier to their PhysioNet profile. The ICU patient group in the MIMIC-III database is known to be diverse, and it includes a wide range of specific biological and clinical traits that can allow the development of precise models based on machine learning algorithms.

Patients were included if they were diagnosed with orthopedic trauma at the time of admission, which was defined as lower or upper limb fractures, spine fractures, and pelvic fractures. The exclusive criteria were as follows: (1) Subjects had an ICU hospitalization duration of less than 24 hours; (3) Subjects died before day 10 after admission (13); (4) Subjects had six or more missing variables; (4) Subjects had spinal cord injury; (5) Subjects had brain injury; (6) Subjects had missing severity of illness scores; (7) Subjects aged less than 18 years.

**Figure 1:**
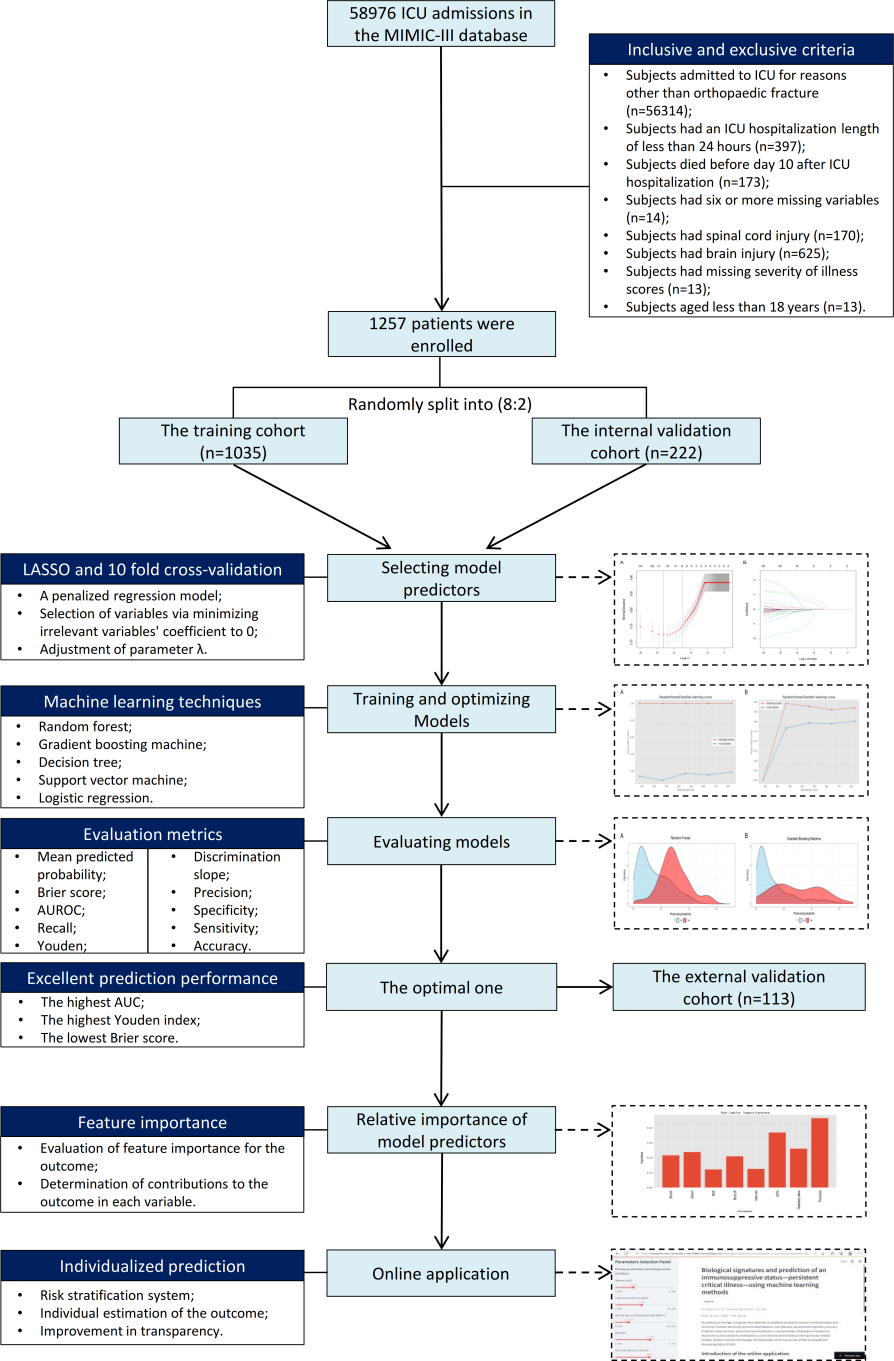
Flow diagram outlining patient enrollment, classification, and the study design.

The protocol for patient’s enrollment and the study’s design are shown in **Figure 1**. To further explain, the enrolled patients were randomly divided into a training cohort (n=1035) and an internal validation cohort (n=222) according to the ratio of 80:20. The model predicting PerCI was trained and optimized based on the training cohort using four machine learning algorithms and a logistic regression model. Internal validation was performed in the internal validation cohort, and external validation was carried out in a multicenter external validation cohort (n=113) from the Xiangya Hospital of Central South University (Changsha) and Shanghai Sixth People’s Hospital Affiliated to Shanghai Jiao Tong University (Shanghai) based on the same inclusive and exclusive criteria.

The Ethics Committee of Shanghai Sixth People’s Hospital Affiliated to Shanghai Jiao Tong University and Hainan Hospital of Chinese PLA General Hospital approved the study and waived the informed consent since all data were anonymous. The study was in accordance with the ethical standards in the 1964 Declaration of Helsinki and its amendments.

### Baseline data and biochemical characteristics

Following literature search and review, forty-one potential risk variables were found and extracted based on the data availability in the MIMIC-III database. These variables were categorized into seven major groups: (1) patient’s demographics, including age and gender; (2) admission laboratory examinations, including albumin (g/dL), creatinine (mg/dL), urea nitrogen (mg/dL), hemoglobin (g/dL), glucose (mg/dL), total calcium (mg/dL), hematocrit (%), sodium (mEq/L), red blood cell distribution width (RDW, %), platelet count (K/uL), and neutrophils (%); (3) admission blood gas analysis, including pH (units), pO2 (mmHg), pCO2 (mmHg), and potassium (mEq/L); (4) admission vital signs, including heart rate (BPM, beats per minute) and respiration rate (BPM); (5) addictions, including tobacco use disorder (%) and alcohol abuse (%); (6) comorbidities, including osteoporosis (%), hypertension (%), respiratory failure (%), dehydration (%), diabetes (%), acute myocardial infarction (%), old myocardial infarction (%), bacteremia (%), pneumonia (%), aortocoronary by pass status (%), obesity (%), atrial fibrillation (%), mitral valve disorders (%), sepsis (%), congestive heart failure (%), ventricular fibrillation (%), and cardiac arrest (%); (7) Severity of illness scores at admission, including the Oxford Acute Severity of Illness Score (OASIS), the Simplified Acute Physiology Score II (SAPS-II), and the Sepsis-related Organ Failure Assessment score (SOFA) (24). In addition, lymphocytes (%) were recorded to investigate the dynamic changes with time from ICU admission to above day 20 after ICU admission. The definition of respiratory failure (**Supplementary Table 1**) and pneumonia (**Supplementary Table 2**) was determined in terms of international classification of diseases-9 (ICD-9) in the MIMIC-III database.

### Outcome: definition of PerCI

The PerCI was defined as having an ICU stay of more than 10 days (8, 11, 13). This newly proposed definition was based on the transition that simple premorbid patient’s characteristics became the predictors of in-hospital mortality rather than the diagnosis and severity of illness at admission and this change occurs after 10 days of ICU admissions (8). This ICU stay length was identified in a series of more than one million patients admitted to 182 ICUs in Australia and New Zealand (8).

### Machine learning-based modelling

Variable selection is essential to achieve the development of prediction model with a large number of covariates. In this study, the model predictors were identified from the potential variables using the Least Absolute Shrinkage and Selection Operator (LASSO) logistic regression method combined with 10-fold cross-validation. As a penalized regression approach, LASSO achieves variable selection through minimizing the comparatively irrelevant variables’ coefficient to 0, thus automatically deleting unnecessary variables. Additionally, the 10-fold cross-validation was adapted to adjust the parameter λ in the LASSO. Selected variables were included as model parameters. Four machine learning algorithms including random forest (25), gradient boosting machine, decision tree, and support vector machine (26) and a logistic regression technique were used to train and optimize models in the training cohort. All models were trained using 5-fold cross-validation and 100 iterations of bootstrapping procedures. Random hyper-parameter search was employed to determine the model parameters such that the area under receiver operating characteristic (AUROC) of the trained model could be maximized. Learning curves of all trained models were presented to evaluate fitting conditions.

### Internal and external validation

The two most important metrics to evaluate the prediction performance of models were discriminative and calibrating ability. Discrimination is the model’s capability to separate patients with PerCI from those without it. Calibration represents the consistency between the observed and predicted probabilities. The prediction performance of the models was validated in the internal validation cohort, and the optimal one was chosen after systematic comparison and then externally validated in a multicenter external validation cohort.

The calculation of AUROC was used to evaluate the discriminative capacity of models, and a model with an AUROC of 0.7 and less than 0.8 is considered as useful and 0.8 or above is regarded as excellent (27). In addition, discrimination slope was also applied in testing the model (28). The slope was determined according to the difference between the mean predicted risk probabilities in patients with and without PerCI (28). Brier score was also used to evaluate the calibrating ability of the prediction models, and this score refers to the squared differences between actual binary outcomes Y and predicted probability p: (Y–p)^2^ (28). A model with a Brier score of above 0.25 is a worthless tool, and lower Brier scores indicates higher calibrating performance of the prediction models. Decision curves were conducted to assess the clinical benefits. In the curve, different threshold probabilities are plotted against net benefits, and treat-for-all and treat-for-none patients’ schedules are considered as two references. The study also analyzed other performance metrics including the accuracy, precision, sensitivity, specificity, and Youden index.

### Risk stratification system

Patients were categorized into two risk groups in terms of the optimal threshold in the optimal prediction model. The low-risk group included patients whose predicted risk probability was below the optimal threshold, while the high-risk group included patients whose predicted risk probability was equal to or greater than the optimal threshold. To compare the actual probabilities between the two groups in the internal and external validation cohorts, subgroup analysis was done.

### Online application

Based on the optimal model, a web-based calculator was established to promote online application. In the calculator, the study presented an introduction to the model, a panel for choosing the variables, and an interface for computing risk probability, and all could be accessed by users through the Internet. The parameters selection panel allows users to pick factors and receive risk probabilities of developing PerCI for specific cases.

### Statistical analysis

Continuous variables with normal distribution were presented as mean with standard deviations and continuous variables without normal distribution were summarized as median with inter-quartile range (IRQ), whereas categorical variables were presented as proportions. Comparison of continuous variables was performed using t test or Wilcoxon rank test, while comparison of categorical variables was performed using Chi-square test or continuous adjusted Chi-square test. Correlation between the model predictors was analyzed using the Pearson correlation analysis, and their Pearson correlation coefficients were visualized. Multiple imputations were employed to address missing data. Feature importance was conducted to evaluate the relative importance of features using machine learning algorithms that best predict outcome, and then the predictors were then ranked by importance. Cumulative hazard curves and the log-rank test were used to compare the difference length of hospital stay between the low- and high-risk groups. Machine learning-based modelling were performed using Python version 3.9.7, and data analysis and visualization were conducted using R programming language (http://www.R-project.org). P values lower than 0.05 was considered as significant.

## Results

### Patient’s baseline and biochemical characteristics

Of all enrolled patients from the MIMIC-III database, 16.0% (201/1257) were identified as in the status of PerCI. The median length of ICU stay was 2.80 (95% CI: 1.76-5.50) days, indicating the majority of patients was discharged within 3 days. The median age was 54.00 (95% CI: 41.00-73.00) years, and more than half of patients were female (58.9%). Hypertension (36.8%) was the most prevalent comorbidity, followed by pneumonia (30.5%), atrial fibrillation (21.7%), congestive heart failure (21.2%), diabetes (20.4%), and respiratory failure (20.0%). Vasopressors were given at admission in 8.4% of cases and mechanical ventilation in 29.9% of cases. More details about patient’s baseline data and biochemical characteristics is summarized in **Table 1**. Among patients from the multicenter cohort, the incidence of PerCI was 27.4% (31/113), and the proportion was significantly greater than that among patients from MIMIC-III. This could be explained by population heterogeneity because patients from the multicenter cohort had a relatively higher proportion of medical comorbidities, a lower level of albumin, and a higher SOFA score (**Supplementary Table 3**), when compared to patients in the MIMIC-III.

**Table 1:** Patient’s demographics and clinical characteristics.

Characteristics	Patients (n=1257)
**Basic demographics**	
Age (median [IQR])	54.00 [41.00, 73.00]
Gender (female/male, %)	740/517 (58.9/41.1)
**Admission laboratory examinations**	
Albumin (g/dL, mean (SD))	3.10 (0.64)
Creatinine (mg/dL, median [IQR])	1.00 [0.80, 1.20]
Urea nitrogen (mg/dL, median [IQR])	18.00 [13.00, 26.00]
Hemoglobin (g/dL, median [IQR])	12.25 [10.60, 13.70]
Glucose (mg/dL, median [IQR])	132.00 [111.00, 162.00]
Total serum calcium (mg/dL, median [IQR])	8.30 [7.80, 8.80]
Hematocrit (%, median [IQR])	36.10 [31.80, 39.80]
Sodium (mEq/L, median [IQR])	139.00 [137.00, 141.00]
RDW (%, median [IQR])	13.70 [13.00, 14.80]
Platelet count (K/uL, median [IQR])	241.00 [184.00, 296.00]
Neutrophils (%, median [IQR])	82.00 [73.72, 88.00]
**Admission blood gas analysis**	
pH (units, median [IQR])	7.36 [7.29, 7.42]
pO_2_ (mmHg, median [IQR])	142.00 [80.00, 248.00]
pCO_2_ (mmHg, median [IQR])	42.72 [37.00, 49.00]
Potassium (mEq/L, median [IQR])	4.00 [3.60, 4.50]
**Admission vital signs**	
Heart rate (BPM, median [IQR])	90.00 [77.00, 104.00]
Respiration rate (BPM, median [IQR])	18.00 [15.00, 22.00]
**Addictions**	
Tobacco use disorder (no/yes, %)	1099/158 (87.4/12.6)
Alcohol abuse (no/yes, %)	1142/115 (90.9/9.1)
**Comorbidities**	
Osteoporosis (no/yes, %)	1146/111 (91.2/8.8)
Hypertension (no/yes, %)	794/463 (63.2/36.8)
Respiratory failure (no/yes, %)	1006/251 (80.0/20.0)
Dehydration (no/yes, %)	1219/38 (97.0/3.0)
Diabetes (no/yes, %)	1000/257 (79.6/20.4)
Acute myocardial infarction (no/yes, %)	1238/19 (98.5/1.5)
Old myocardial infarction (no/yes, %)	1182/75 (94.0/6.0)
Bacteremia (no/yes, %)	1201/56 (95.5/4.5)
Pneumonia (no/yes, %)	873/384 (69.5/30.5)
Aortocoronary by pass status (no/yes, %)	1197/60 (95.2/4.8)
Obesity (no/yes, %)	1196/61 (95.1/4.9)
Atrial fibrillation (no/yes, %)	984/273 (78.3/21.7)
Mitral valve disorders (no/yes, %)	1201/56 (95.5/4.5)
Sepsis (no/yes, %)	1142/115 (90.9/9.1)
Congestive heart failure (no/yes, %)	990/267 (78.8/21.2)
Ventricular fibrillation (no/yes, %)	1252/5 (99.6/0.4)
Cardiac arrest (no/yes, %)	1221/36 (97.1/2.9)
**Severity of illness score at admission**	
OASIS (median [IQR])	31.00 [26.00, 37.00]
SAPS-II (median [IQR])	31.00 [21.00, 40.00]
SOFA (median [IQR])	3.00 [1.00, 5.00]

IRQ, inter-quartile range; SD, standard deviation; RDW, red blood cell distribution width; BPM, beats per minute; OASIS, the Oxford Acute Severity of Illness Score; SAPS, the Simplified Acute Physiology Score; SOFA, the Sepsis-related Organ Failure Assessment score.

### Dynamic changes of lymphocytes between patients with and without PerCI

**Figure 2:**
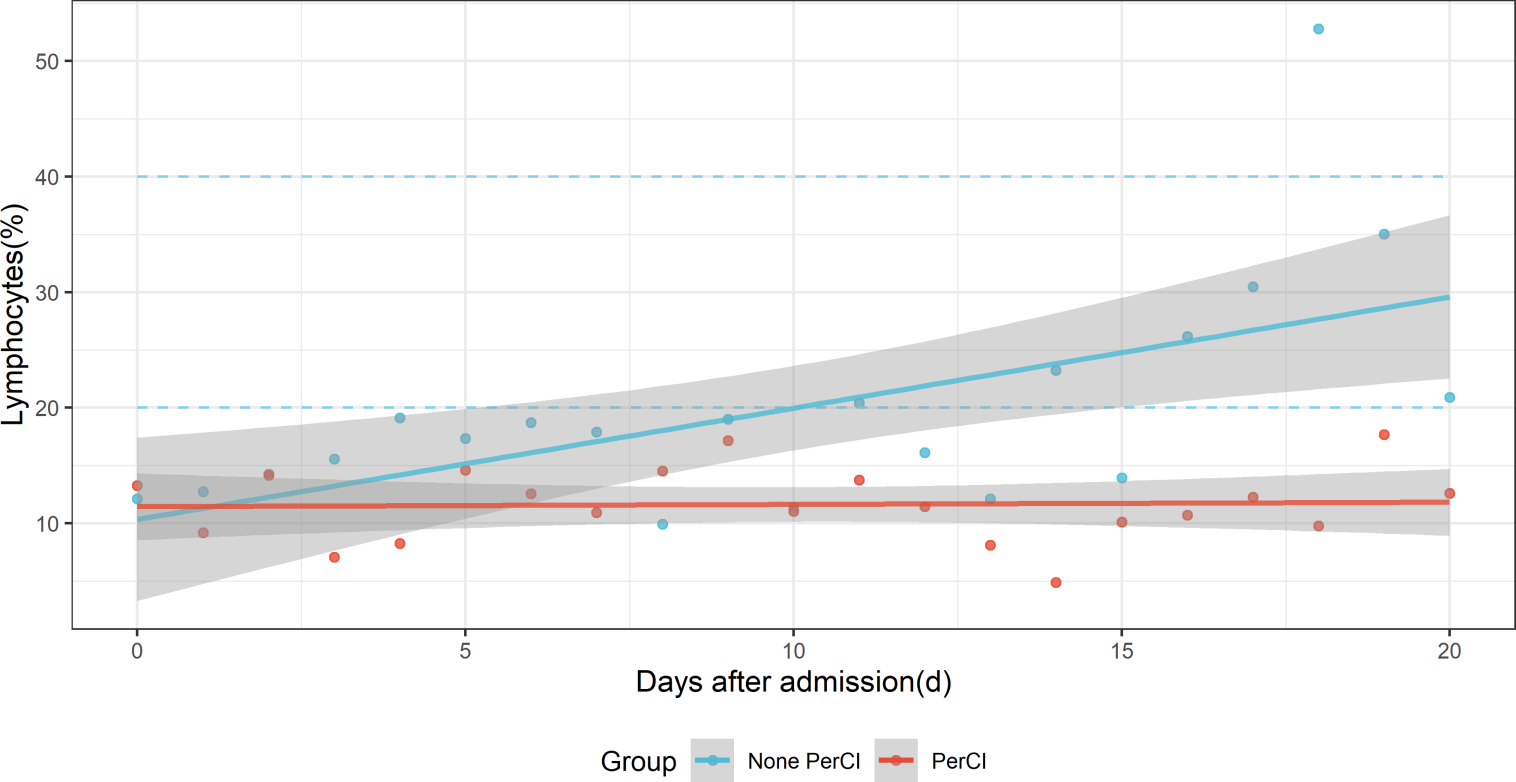
Dynamic changes of the mean level of lymphocytes (%) with time after ICU admission between patients with (red dots) and without (sky-blue dots) persistent critical illness. The blue-sky dotted line indicates normal reference range. The red and sky-blue dots are fitted by smooth lines respectively.

At admission, the level of lymphocytes (%) was similar between patients with (9.30, [95% CI: 5.60-16.90]) and without (9.25, [95% CI: 6.00-15.10]) PerCI (P=0.582, Wilcoxon rank test, **Supplementary Table 4**). However, the means of lymphocytes were consistently below the normal reference range (20%-40%) across the time among PerCI patients, with the majority of those numbers hovering around 10.0%, whereas the level of lymphocytes continued to increase among patients without PerCI, and it began to be in the normal range at day 10 after ICU admission (**Figure 2** and **Supplementary Figure 1**).

### A comparison of biochemical and clinical characteristics between patients with and without PerCI

Subgroup analysis demonstrated that patients with PerCI were in a more serious health condition since patients with PerCI tended to have worse nutritional status, more electrolyte imbalance, more infection-related comorbidities, and more severe illness scores (**Table 2**). More specifically, patients with PerCI have the lower albumin (P<0.001), higher glucose (P<0.001), lower total calcium (P<0.001), higher sodium (P=0.001), lower blood pH (P<0.001), higher heart rate (P=0.001), higher respiratory failure (P<0.001), higher bacteremia (P<0.001), higher pneumonia (P<0.001), lower aortocoronary by pass status (P=0.028), higher sepsis (P<0.001), higher ventricular fibrillation (P=0.038), higher cardiac arrest (P<0.001), higher OASIS score (P<0.001), higher SAPS-II score (P<0.001), and higher SOFA score (P<0.001), as compared to patients without PerCI. Similar findings were obtained in the multicenter cohort (**Supplementary Table 5**).

**Table 2:** A comparison of clinical characteristics between patients with and without persistent critical illness.

Characteristics	Persistent critical illness		P
	No	Yes	
n	1056	201	
**Basic demographics**			
Age (median [IQR])	54.00 [41.00, 73.00]	54.00 [43.00, 73.00]	0.720
Gender (female/male, %)	618/438 (58.5/41.5)	122/79 (60.7/39.3)	0.620
**Admission laboratory examinations**			
Albumin (g/dL, mean (SD))	3.15 (0.62)	2.86 (0.70)	<0.001
Creatinine (mg/dL, median [IQR])	1.00 [0.80, 1.20]	1.00 [0.80, 1.30]	0.247
Urea nitrogen (mg/dL, median [IQR])	18.00 [13.00, 26.00]	18.00 [13.00, 24.00]	0.865
Hemoglobin (g/dL, median [IQR])	12.30 [10.65, 13.70]	11.90 [10.50, 13.50]	0.139
Glucose (mg/dL, median [IQR])	130.00 [110.00, 159.00]	148.00 [123.00, 193.00]	<0.001
Total serum calcium (mg/dL, median [IQR])	8.40 [7.90, 8.80]	8.10 [7.40, 8.70]	<0.001
Hematocrit (%, median [IQR])	36.30 [32.00, 39.80]	35.10 [31.10, 39.20]	0.082
Sodium (mEq/L, median [IQR])	139.00 [137.00, 141.00]	140.00 [138.00, 142.00]	0.001
RDW (%, median [IQR])	13.70 [13.00, 14.83]	13.70 [13.00, 14.70]	0.730
Platelet count (K/uL, median [IQR])	244.00 [185.00, 298.50]	233.00 [182.00, 279.00]	0.163
Neutrophils (%, median [IQR])	82.00 [73.42, 88.00]	81.10 [74.60, 88.20]	0.859
**Admission blood gas analysis**			
pH (units, median [IQR])	7.36 [7.29, 7.42]	7.33 [7.22, 7.40]	<0.001
pO_2_ (mmHg, median [IQR])	143.52 [83.14, 250.34]	119.00 [67.00, 227.00]	0.070
pCO_2_ (mmHg, median [IQR])	42.45 [37.00, 49.00]	43.00 [38.00, 51.00]	0.117
Potassium (mEq/L, median [IQR])	4.00 [3.60, 4.51]	3.90 [3.60, 4.30]	0.068
**Admission vital signs**			
Heart rate (BPM, median [IQR])	90.00 [76.00, 103.00]	94.00 [81.00, 111.00]	0.001
Respiration rate (BPM, median [IQR])	18.00 [15.00, 22.00]	18.00 [15.00, 22.00]	0.241
**Addictions**			
Tobacco use disorder (no/yes, %)	919/137 (87.0/13.0)	180/21 (89.6/10.4)	0.382
Alcohol abuse (no/yes, %)	959/97 (90.8/9.2)	183/18 (91.0/9.0)	1.000
**Comorbidities**			
Osteoporosis (no/yes, %)	961/95 (91.0/9.0)	185/16 (92.0/8.0)	0.735
Hypertension (no/yes, %)	672/384 (63.6/36.4)	122/79 (60.7/39.3)	0.476
Respiratory failure (no/yes, %)	896/160 (84.8/15.2)	110/91 (54.7/45.3)	<0.001
Dehydration (no/yes, %)	1021/35 (96.7/3.3)	198/3 (98.5/1.5)	0.247
Diabetes (no/yes, %)	846/210 (80.1/19.9)	154/47 (76.6/23.4)	0.302
Acute myocardial infarction (no/yes, %)	1040/16 (98.5/1.5)	198/3 (98.5/1.5)	1.000
Old myocardial infarction (no/yes, %)	991/65 (93.8/6.2)	191/10 (95.0/5.0)	0.628
Bacteremia (no/yes, %)	1019/37 (96.5/3.5)	182/19 (90.5/9.5)	<0.001
Pneumonia (no/yes, %)	802/254 (75.9/24.1)	71/130 (35.3/64.7)	<0.001
Aortocoronary by pass status (no/yes, %)	999/57 (94.6/5.4)	198/3 (98.5/1.5)	0.028
Obesity (no/yes, %)	1007/49 (95.4/4.6)	189/12 (94.0/6.0)	0.532
Atrial fibrillation (no/yes, %)	831/225 (78.7/21.3)	153/48 (76.1/23.9)	0.473
Mitral valve disorders (no/yes, %)	1009/47 (95.5/4.5)	192/9 (95.5/4.5)	1.000
Sepsis (no/yes, %)	976/80 (92.4/7.6)	166/35 (82.6/17.4)	<0.001
Congestive heart failure (no/yes, %)	835/221 (79.1/20.9)	155/46 (77.1/22.9)	0.598
Ventricular fibrillation (no/yes, %)	1054/2 (99.8/0.2)	198/3 (98.5/1.5)	0.038
Cardiac arrest (no/yes, %)	1034/22 (97.9/2.1)	187/14 (93.0/7.0)	<0.001
**Severity of illness score at admission**			
OASIS (median [IQR])	31.00 [25.00, 37.00]	34.00 [30.00, 40.00]	<0.001
SAPS-II (median [IQR])	30.00 [20.00, 40.00]	35.00 [27.00, 42.00]	<0.001
SOFA (median [IQR])	3.00 [1.00, 5.00]	5.00 [3.00, 7.00]	<0.001

IRQ, inter-quartile range; SD, standard deviation; RDW, red blood cell distribution width; BPM, beats per minute; OASIS, the Oxford Acute Severity of Illness Score; SAPS, the Simplified Acute Physiology Score; SOFA, the Sepsis-related Organ Failure Assessment score.

### Establishment of machine learning-based models

The 1257 enrolled patients were randomly divided into a training cohort (n=1035) and a validation cohort (n=222). In the training group, the LASSO logistic regression along with the 10-fold cross-validation identified eight predictors, including admission albumin, serum calcium, RDW, blood pH, heart rate, respiratory failure, pneumonia, and SOFA. Binomial deviance curve presented the optimal values at the minimum criteria and the one standard error of the minimum criteria (**Supplementary Figure 2A**), and LASSO coefficient profiles of all variables were shown (**Supplementary Figure 2B**). Correlation between the eight variables was analyzed, all correlation coefficients were less than 0.40, and only two of twenty-eight coefficients were above 0.30, all indicating no serious collinearity existed between the eight variables (**Supplementary Figure 3**). Thus, the eight variables were all included for model training and optimizing. Four machine learning algorithms (random forest, gradient boosting machine, decision tree, and support vector machine) and a logistic regression technique were trained on the 1035-patient training cohort for prediction of developing PerCI. Using randomized search with cross validation, the optimal parameters could be determined, and **Supplementary Table 6** summaries the full super-parameters of all algorithms. The learning curves for the random forest (**Supplementary Figures 4A, B**), gradient boosting machine (**Supplementary Figures 4C, D**), decision tree (**Supplementary Figures 5A, B**), support vector machine (**Supplementary Figures 5C, D**), and logistic regression (**Supplementary Figures 5E, F**) denoted that underfitting and overfitting were considerably avoided after searching for optimal super-parameters in each algorithm.

In addition, SHAP (SHapley Additive exPlanations) elucidated that a higher proportion of pneumonia and respiratory failure, a higher SOFA score and heart rate, and lower RDW, albumin, serum calcium, and blood pH were risk contributors for developing PerCI (**Supplementary Figure 6**).

### Internal and external validation

**Figure 3:**
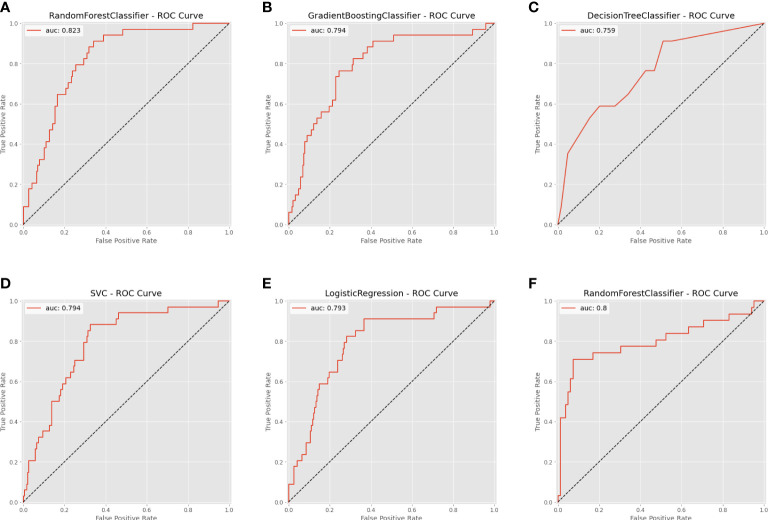
Area under the receiver operating characteristic curve (AUROC) for the machine learning-based models. **(A)** Random Forest for internal validation; **(B)** Gradient Boosting Machine; **(C)** Decision Tree; **(D)** Support Vector Machine; **(E)** Logistic Regression; **(F)** Random Forest for external validation.

**Figure 4:**
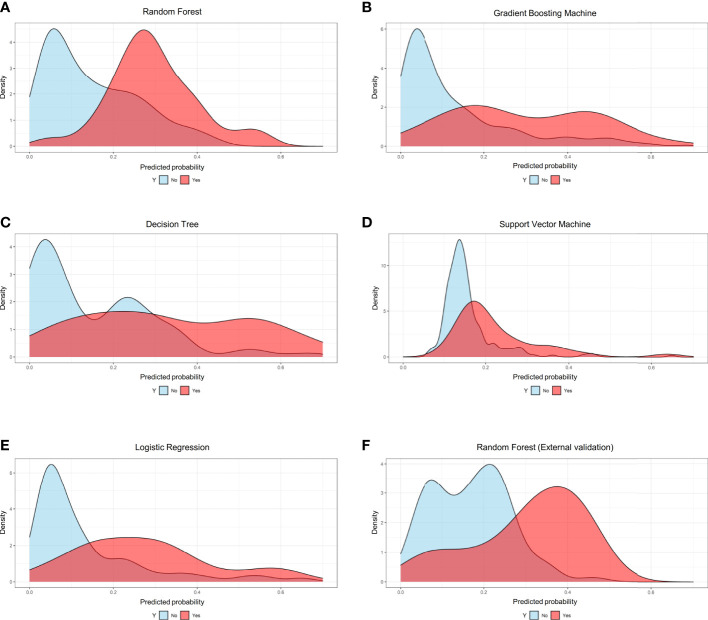
Probability curves for the machine learning-based models. **(A)** Random Forest for internal validation; **(B)** Gradient Boosting Machine; **(C)** Decision Tree; **(D)** Support Vector Machine; **(E)** Logistic Regression; **(F)** Random Forest for external validation.

**Figure 5:**
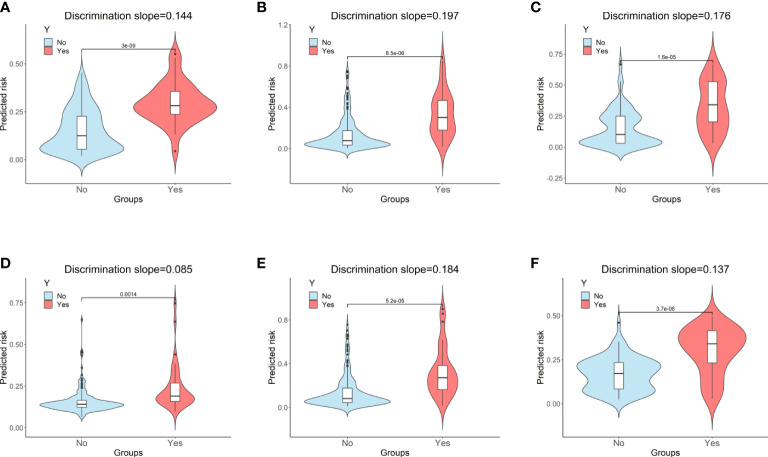
Violin plots of predicted probability with (red) and without (sky blue) the outcome. **(A)** Random Forest for internal validation; **(B)** Gradient Boosting Machine; **(C)** Decision Tree; **(D)** Support Vector Machine; **(E)** Logistic Regression; **(F)** Random Forest for external validation. “Y” indicates the outcome; “No” indicates patients without persistent critical illness; “Yes” indicates patients with persistent critical illness.

**Figure 6:**
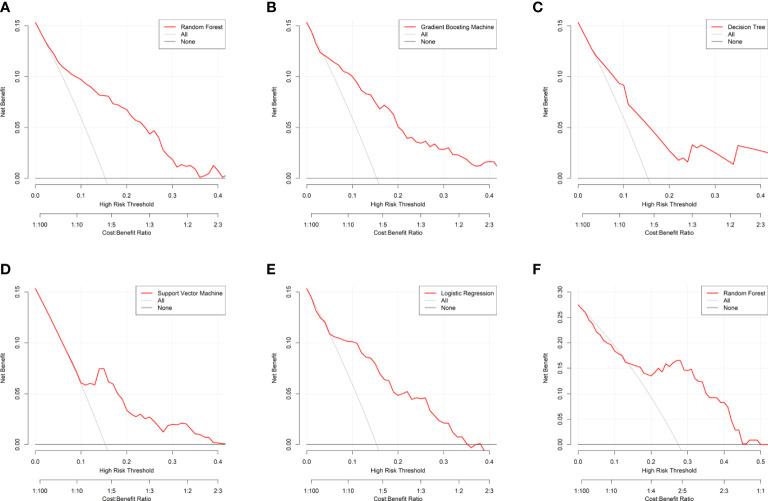
Decision curve analysis for the machine learning-based models. **(A)** Random Forest for internal validation; **(B)** Gradient Boosting Machine; **(C)** Decision Tree; **(D)** Support Vector Machine; **(E)** Logistic Regression; **(F)** Random Forest for external validation. The horizontal black line is treated-for-none scheme, and the solid gray line is treated-for-all scheme.

Based on the internal validation (**Figure 3**), the highest performing model was the random forest, with an AUROC of 0.823 (95% CI: 0.757-0.889), Youden index of 1.571, and Brier score of 0.107 (**Table 3**). External validation also showed the AUROC could be up to 0.800 (95% CI: 0.688-0.912), Youden index was 1.637, and accuracy was up to 0.867. As expected, the prediction performance of random forest also outperformed SOFA (AUROC: 0.631), OASIS (AUROC: 0.659), and SAPS II (AUROC: 0.578) (**Supplementary Figure 7**). Probability curves demonstrated that the random forest was favorable, with large separation and small overlap between patients with and without PerCI in the both internal and external validation cohort (**Figure 4**). **Figure 5** depicts that the difference of predicted probability between patients with and without PerCI was all significant no matter in the internal validation cohort including the four machine learning algorithms and a logistic regression technique or in the external validation cohort. Decision curve analysis demonstrated that all models, particular the random forest, had favorable clinical usefulness (**Figure 6**).

**Table 3:** Prediction performance of machine learning algorithms for predicting persistent critical illness among patients with orthopedic fracture.

Measures	Internal validation					External validation
	Random forest	Gradient boosting machine	Decision tree	Support vector machine	Logistic regression	
Actual probability	0.153	0.153	0.153	0.153	0.153	0.270
Mean predicted probability	0.173	0.170	0.180	0.171	0.169	0.206
Brier score	0.107	0.112	0.113	0.116	0.114	0.164
AUROC (95%CI)	0.823 (0.757-0.889)	0.794 (0.711-0.876)	0.759 (0.671-0.847)	0.794 (0.716-0.871)	0.793 (0.712-0.875)	0.800 (0.688-0.912)
Discrimination slope	0.144	0.197	0.176	0.085	0.184	0.137
Specificity	0.660	0.755	0.489	0.676	0.633	0.927
Sensitivity	0.912	0.765	0.912	0.882	0.912	0.710
Precision	0.326	0.361	0.244	0.330	0.310	0.786
Youden	1.571	1.520	1.401	1.558	1.545	1.637
Accuracy	0.698	0.757	0.554	0.707	0.676	0.867
Threshold	0.184	0.174	0.098	0.154	0.117	0.279

AUROC, Area under the receiver operating curve; CI, Confident interval.

### Feature importance

**Figure 7:**
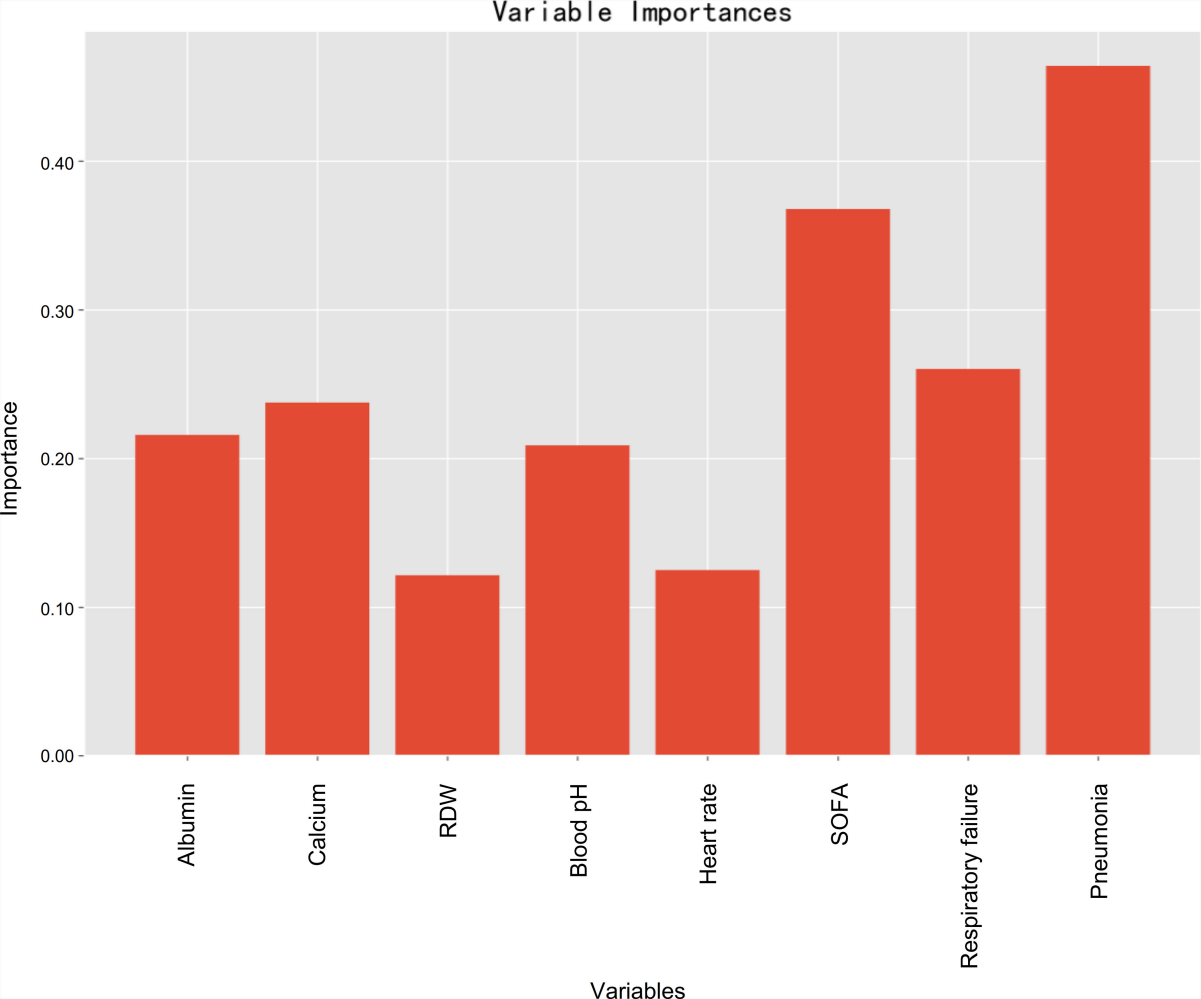
Feature importance of model predictors in terms of the random forest algorithm.

Variable importance estimations were employed to determine the relative feature importance in terms of the final machine learning model (random forest), and it found that pneumonia, followed by SOFA, was the most important variable for prediction of developing PerCI (**Figure 7**), such that an increase in either pneumonia or SOFA would be more likely to result in PerCI. Gradient boosting machine (**Supplementary Figure 8**) and decision tree (**Supplementary Figure 9**) both also confirmed that pneumonia was the most important variable based on analysis of feature importance. Additionally, other important variables included respiratory failure, serum calcium, albumin, blood pH, heart rate, and RDW in a descending sequence of importance.

### Risk stratification system

**Figure 8:**
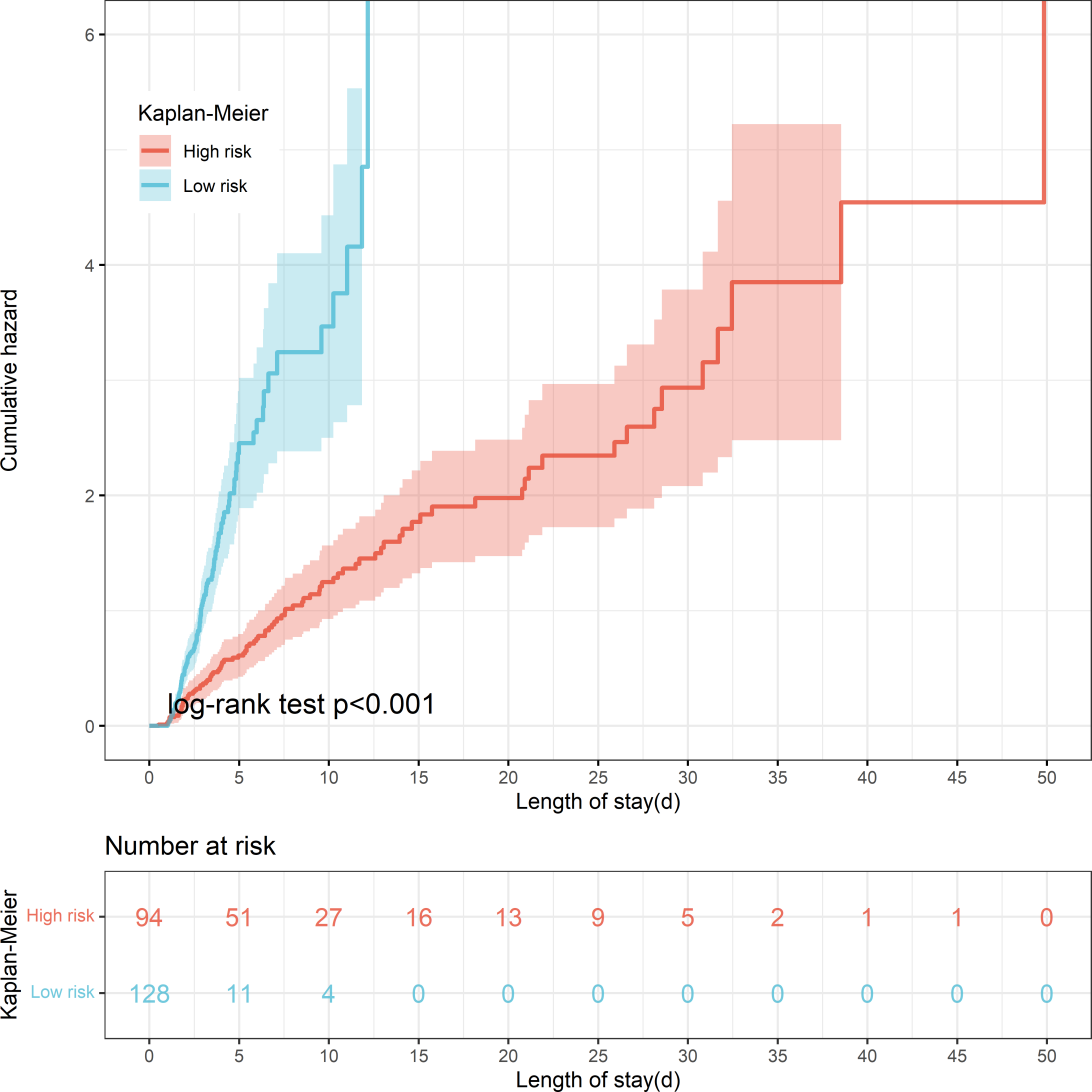
Kaplan-Meier curve shows cumulative hazard stratified by risk stratification system (P < 0.001, Log-rank test). “Sky bule” indicates patients in the low-risk group; “Red” indicates patients in the high-risk group.

The threshold was 18.4% in the final machine learning model and it was used to conduct risk stratification. Patients were stratified into two risk groups: Patients with an anticipated risk probability of less than 18.4% were categorized into the low-risk group, whereas patients with an anticipated risk probability of 18.4% or above were categorized into the high-risk group (**Table 4**). Patients who were classified into the high-risk group were slightly above 10-time more vulnerable to develop PerCI than those in the low-risk group in the internal validation cohort (P<0.001). Similar results were found in the external validation cohort and the number was more than 3 times (P=0.001). Cumulative hazard curve further demonstrated that patients in the high-risk groups had a significant longer length of ICU stay than patients in the low-risk groups in both the internal (**Figure 8**) and external (**Supplementary Figure 10**) validation cohorts. In addition, the mean percent of lymphocytes among patients in the high-risk group of developing PerCI remain stable and low at about 10%, while the level of lymphocytes among patients in the low-risk group continued to grow after admission (**Supplementary Figures 11** and **12**). The above results suggested that this risk stratification system performed well to distinguish patients at high and low risk of developing the outcome.

**Table 4:** Risk stratification of patients in the validation cohorts based on the final machine learning model.

Risk group	Patients	Likelihood of PerCI		P ^a^
		Predicted	Actual	
**Internal validation cohort**				
Low-risk group (<18.40%)	128	8.44%	3.13% (4/128)	<0.001
High-risk group (≥18.40%)	94	29.44%	31.91% (30/94)	
**External validation cohort**				
Low-risk group (<18.40%)	53	10.14%	13.21% (7/53)	0.001
High-risk group (≥18.40%)	60	29.77%	40.00% (24/60)	

a indicates A comparison of actual probability between the low-risk and high-risk groups in the internal and external validation cohorts using the Chi-Square test.

PerCI, persistent critical illness.

### Online application

**Figure 9:**
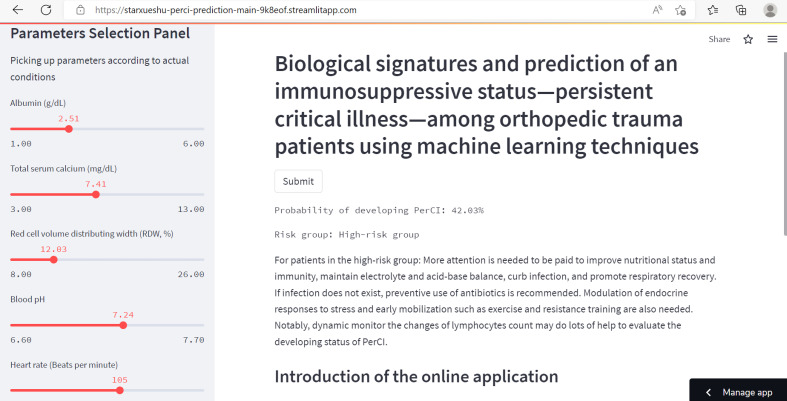
The partial interface of the online application. The left part shows a panel on selection of parameters. The right part shows predicted probability of developing PerCI, individualized preventive strategies, risk stratification system, and introduction of the model.

Based on the final machine learning model, the study developed an easy-to-use web-based calculator for PerCI prediction (**Figure 9**), and this calculator is accessible at https://starxueshu-perci-prediction-main-9k8eof.streamlitapp.com/. The online application contains of a panel for variables selection, a section for introducing the model, and a calculating interface. By picking up characteristics for specific patients, the predicted risk probability of developing PerCI could be immediately obtained. At the same time, risk stratification system and individual recommendation of preventive and therapeutic strategies were also presented for users.

## Discussion

This study examined the underlying biochemical signatures and pathophysiology driving PerCI, and further proposed an accurate model based on machine learning algorithms. The study found that low nutritional status, electrolyte imbalance, infection-related comorbidities, and severe illness scores at admission were risk contributors for developing PerCI among orthopedic trauma patients. To elaborate, lower RDW, albumin, serum calcium, and blood pH, a higher SOFA score and heart rate, and a higher proportion of pneumonia and respiratory failure were significantly associated with more PerCI according to the analysis of LASSO and SHAP. These finding suggested that improving nutritional status and immunity, maintaining electrolyte and acid-base balance, curbing infection, and promoting respiratory function were early warranted to prevent the onset of PerCI. More importantly, the present study examined the dynamic changes of lymphocytes over time following admission, and it suggested that dynamic monitor of lymphocytes count could provide a powerful indicator of the development of PerCI.

In recent years, the population of PerCI was evidently growing mainly due to the improvements of modern techniques (19, 29, 30). Such patients required far longer time span of multi-organ support along with many other advanced life support equipment and consumed an excessive amount of health resources. In the present study, 15.99% of patients developed PerCI among orthopedic trauma patients in the MIMIC-III database (201/1257), which was consistent with other studies with an incidence of 20.00% among trauma patients (15). However, in the multicenter cohort, the incidence was 27.43%, and the number was significantly higher than that in the MIMIC-III database. This difference could be explained by population heterogeneity since patients from the multicenter cohort were in more serious health condition.

Studies have revealed the process by which patients transit from a critical acute disease to this new status (31). Immunosuppression (3), persistent inflammation (17), and endocrine dysfunction (18) were proposed as a mechanistic framework in which to describe the characteristics of PerCI. Key drivers of this immunosuppression and persistent inflammation are myeloid-derived suppressor cells that are able to influence almost every cell of host innate and adaptive immunity (3). A vicious cycle of immune dyscrasia between hematopoietic stem cells and myeloid-derived suppressor cells exists to promote PerCI (19). Previous studies have focused on the timing of onset and the potential biochemical signatures of PerCI. Nonetheless to the author’s knowledge, no useful prognostic strategies have been brought up to identify the vulnerable individuals who were subject to PerCI. As a consequence, most of the patients were confirmed of PerCI based on expert opinions that requires certain amount of time (31). Under such circumstance, it was difficult to make beneficial interventions in advance.

To further establish an accurate prediction model, this study introduced machine learning algorithms and focused on patients with orthopedic trauma. In terms of selecting potential model predictors, LASSO equipped with 10-fold cross-validation were applied in the study. LASSO is a penalized regression method that can minimize the residual sum of squares subjected to the sum of absolute value of variable coefficients being less than a constant, and it involves in great reliability in selection of most relevant variables from a large number of covariates (32). The estimates of the regression coefficients can be sparsely reduced to 0, which enables LASSO to automatically remove unnecessary covariates (33). At last, eight variables that were identified by LASSO were served as model predictors. These identified variables were mostly modifiable, and thus this suggested that some measures to increase albumin and RDW levels, avoid hypocalcemia and tachycardia, regulate blood pH, and control or treat respiratory failure and infection such as sepsis and pneumonia would be of great help in the prevention and management of PerCI. Previous studies have confirmed several variables were associated with this status. For example, Jeffcote et al. (10) reported that respiratory, septic, and neurosurgical admission diagnoses and ventilator-associated pneumonia were closely linked with PerCI among ICU patients, and this was in line with the present study. Darvall et al. (34) concluded that PerCI had an inclination to be resulted from acquired complication after ICU admission, such as new sepsis, and was mostly irrelevant to the original illness according to a matched-pair analysis. In addition, patients with PerCI were more likely to require ICU supports and suffer from ICU-acquired weakness or delirium. Viglianti et al. (14) investigated how hospital-level characteristics affected the development of PerCI in 100 hospitals encompassing 153,512 ICU hospitalizations and found that hospitals with higher risk- and reliability-adjusted 30 days mortality had a higher probability of persistently critically ill patients. Nomellini et al. (35) summarized that advanced age, comorbidities, severe injury, septic shock, and malnutrition were associated with the development of PerCI. Horn et al. (20) also elucidated that chronic critical ill patients tended to be older and have a higher BMI compared to rapid recovery patients after severe trauma in a series of 60 cases, but these factors were nonmodifiable.

Regarding laboratory examinations, previous studies pointed out that elevated urea-to-creatinine ratio might provide a biochemical marker of PerCI after major trauma (13) and among sepsis patients (36). Zhang et al. (36) also found that PerCI patients had significantly greater illness severity score, and lower albumin and hemoglobin than those without it, but C-reactive protein was not significantly different between the patients with and without the status among patients with sepsis from the eICU database. Darden et al. (22) found that IL-10 and IP-10 on day four after admission could improve PerCI prediction among patients with surgical sepsis, but the improved effectiveness was only minimal. The present study further added new valuable supplements to current literature, and it demonstrated that a lower level of albumin, calcium, RDW, and blood pH might be important drivers of PerCI. The level of serum albumin could decline considerably even in early stage of critical illness patients, which also suggested the favorable effects of timely supplying albumin (37). In addition, Stortz et al. (38) found that PerCI patients tended to have a greater incidence of secondary infections and persistent abnormal biomarkers of impaired host immunity. Of note, the present study found that patients with PerCI were continuing to be in an immunosuppressive status after analyzing the level of lymphocytes, while in patients without PerCI, lymphocytes gradually elevated and began to be in the normal range at day 10 after ICU admission. Thus, dynamically examining the level of lymphocytes would be considerably beneficial to understand the development of PerCI. To the author’s knowledge, this finding was the first to systematically to depict the changes of lymphocytes over time following ICU admission. Given these findings, understanding these specific biochemical characteristics was greatly conducive to reduce the likelihood of developing this status.

The validation of the final optimal machine learning-based model was performed in the internal validation cohort and the multicenter external validation cohort, both of which reflected favorable discrimination and calibration. These findings indicated that the prediction model was readily applicable in ICU settings. Patients were divided into two risk groups based on the threshold: the low and high-risk groups. Patients in the high-risk group were above 10-time greater of odds to develop PerCI than patients in the low-risk group, and thus special attentions and beneficial interventions, such as, early mobilization, antibiotics, fluid resuscitation, as well as adequate supply of caloric (19), were early warranted among those patients in the high-risk groups. In a systematic review, in-hospital and post-discharge recommendations were summarized to prevent PerCI (19). The in-hospital suggestions included (1) optimal early healthcare, such as antibiotics, fluid resuscitation, vasopressors, and source control, (2) management of pain, agitation, and delirium, (3) early mobilization, such as exercise and resistance training, and (4) modulation of endocrine responses to stress and early enteral nutrition (35). The post-discharge recommendations included (1) identification of functional disability and impairments in swallowing and mental health, (2) review and adjust long-term medications to avoid medication errors, and (3) prevention of common causes of health deterioration, such as infection, acute heart and/or renal failure, and respiratory diseases.

### Limitations

Limitations of this study should be acknowledged. First of all, this is a retrospective observational study, and selection bias may not be avoided. Notably, MIMIC-III database is widely used data sources with high quality, which is able to support our development of the prediction model substantially. Secondly, some variables such as the level of IL-10 and IP-10 (22) were not considered in our study due to unavailability of these variables, leading to confounding bias, but this study analyzed up to forty-one potential variables including basic demographics, admission vital signs, admission laboratory tests, admission blood gas, addictions, severity of illness score, and comorbidities, indicating that the analyzed variables were comprehensive. Thirdly, the measure of lymphocytes may cannot fully reflect the extent of immunity, and the measurement of other immunological mediators would allow more comprehensive analysis. Therefore, despite the fact the model have favorable discrimination and calibration, future investigations on extensive external are still warranted.

## Conclusions

Patients with PerCI typically remain in an immunosuppressive status, but those without PerCI gradually regain normal immunity. The dynamic changes of lymphocytes can be a reliable biomarker for PerCI. This work developed a reliable model that may be helpful in improving early diagnosis and targeted intervention of PerCI. Beneficial interventions, such as improving nutritional status and immunity, maintaining electrolyte and acid-base balance, curbing infection, and promoting respiratory recovery, are early warranted to prevent the onset of PerCI, especially among patients in the high-risk group and those with a continuously low level of lymphocytes.

## Data availability statement

Publicly available datasets were analyzed in this study. This data can be found here: The data from MIMIC-III were available at https://mimic.mit.edu/. The multicenter data in the study are available upon reasonable request to the corresponding authors.

## Ethics statement

The PhysioNet approved authors who met requirements to access to the MIMIC-III database. The author completed all training courses including the Human Research, Data or Specimens Only Research, and 1-Basic Course, and acquired the data user requirements (Record ID: 32128436). The Ethics Committee of Shanghai Sixth People’s Hospital Affiliated to Shanghai Jiao Tong University and Hainan Hospital of Chinese PLA General Hospital approved the study and waived the informed patient’s consent since all data were anonymous. Written informed consent for participation was not required for this study in accordance with the national legislation and the institutional requirements.

## Author contributions

ML, ZH, and SW performed study concept and design. ZH, SW, and YS did the acquisition of subjects and/or clinical data. ML, SW, and CG conducted analysis and interpretation of data. XZ, TH, and FL supervised the study. FL provided funding. All authors assisted with reading, editing, and approving the manuscript.

## Funding

This study was funded by Hainan Province Clinical Medical Center. The sponsors were not involved in any aspect of the project or in preparation of the manuscript.

## Conflict of interest

Author CG was employed by the company Hengpu Yinuo Beijing Technology Co., Ltd, China.

The remaining authors declare that the research was conducted in the absence of any commercial or financial relationships that could be construed as a potential conflict of interest.

## Publisher’s note

All claims expressed in this article are solely those of the authors and do not necessarily represent those of their affiliated organizations, or those of the publisher, the editors and the reviewers. Any product that may be evaluated in this article, or claim that may be made by its manufacturer, is not guaranteed or endorsed by the publisher.

## Supplementary material

Supplementary Figure 1 Line chart for the dynamic changes of the mean level of lymphocytes (%) with time after ICU admission between patients with (red line) and without (sky-blue line) persistent critical illness. The black dotted line indicates normal reference range.

Supplementary Figure 2 Selection of model predictors using the least absolute shrinkage and selection operator (LASSO) logistic regression model combined with 10-fold cross-validation. (**A**) Selection of appropriate parameters (λ) from the LASSO method by ten-fold cross-validation and minimum criteria. Binomial deviance curve was plotted against log (λ). Dotted vertical lines were drawn at the optimal values by the minimum criteria and the one standard error of the minimum criteria; (**B**) LASSO coefficient profiles of all variables. Coefficient curve was plotted against the log lambda sequence. A vertical line was presented at the value.

Supplementary Figure 3 Correlation matrix for model predictors. Panels in the lower left show scatted diagrams between the eight model predictors, and smooth lines (red) were fitted. Panels in the upper right show correlation coefficients between the eight predictors. Panels in the diagonal line show histograms of each predictor. * indicates P<0.05, ** indicates P<0.01, *** indicates P<0.001.

Supplementary Figure 4 Learning curves for machine learning. **(A)** Random Forest; (**B**) Gradient Boosting Machine.

Supplementary Figure 5 Learning curves for machine learning. **(A)** Decision Tree; **(B)** Support Vector Machine; **(C)** Logistic Regression.

Supplementary Figure 6 Analysis of model predictors in terms of SHapley Additive exPlanations. It presents how the Shapley value distribute in each predictor. The color represents the eigenvalue, and red indicates high value (risk contributor to the outcome) and blue indicates low value (protective contributor to the outcome) in the study.

Supplementary Figure 7 Area under the receiver operating characteristic curve (AUROC) for a comparison among the random forest algorithm, the Oxford Acute Severity of Illness Score (OASIS), the Simplified Acute Physiology Score II (SAPS-II), and the Sepsis-related Organ Failure Assessment score (SOFA).

Supplementary Figure 8 Feature importance of model predictors in terms of the gradient boosting machine algorithm.

Supplementary Figure 9 Feature importance of model predictors in terms of the decision tree algorithm.

Supplementary Figure 10 Kaplan-Meier curve shows cumulative hazard stratified by risk stratification system in the external validation cohort (P=0.024, Log-rank test). “Sky bule” indicates patients in the low-risk group; “Red” indicates patients in the high-risk group.

Supplementary Figure 11 Line chart for the dynamic changes of the mean level of lymphocytes (%) with time after ICU admission between patients in the low-risk group (sky-blue line) and patients in the high-risk group (red line). The black dotted line indicates normal reference range.

Supplementary Figure 12 Dynamic changes of the mean level of lymphocytes (%) with time after ICU admission between patients in the low-risk group (sky-blue dots) and patients in the high-risk group (red dots). The blue-sky dotted line indicates normal reference range. The red and sky-blue dots are fitted by smooth lines respectively.
