# Supplementary material for: Biological signatures and prediction of an immunosuppressive status—persistent critical illness—among orthopedic trauma patients using machine learning techniques
Source: Front Immunol. 2022 Oct 17;13:979877. doi: 10.3389/fimmu.2022.979877 (PMC9620964; doi:10.3389/fimmu.2022.979877)
Supplement: Supplementary file 1 [file Table_1.docx]

| **Supplementary table 1 \|** Definition of respiratory failure in terms of ICD-9. | | |
| --- | --- | --- |
| **ICD-9** | **Short title** | **Long title** |
| 51851 | Ac resp flr fol trma/srg | Acute respiratory failure following trauma and surgery |
| 51853 | Ac/chr rsp flr fol tr/sg | Acute and chronic respiratory failure following trauma and surgery |
| 51881 | Acute respiratry failure | Acute respiratory failure |
| 51883 | Chronic respiratory fail | Chronic respiratory failure |
| 51884 | Acute & chronc resp fail | Acute and chronic respiratory failure |
| ICD-9, international classification of diseases-9. | | |
