# Supplementary material for: Biological signatures and prediction of an immunosuppressive status—persistent critical illness—among orthopedic trauma patients using machine learning techniques
Source: Front Immunol. 2022 Oct 17;13:979877. doi: 10.3389/fimmu.2022.979877 (PMC9620964; doi:10.3389/fimmu.2022.979877)
Supplement: Supplementary file 2 [file Table_2.docx]

| **Supplementary table 2** **\|** Definition of pneumonia in terms of ICD-9. | | |
| --- | --- | --- |
| **ICD-9** | **Short title** | **Long title** |
| 4829 | Bacterial pneumonia NOS | Bacterial pneumonia, unspecified |
| 4870 | Influenza with pneumonia | Influenza with pneumonia |
| 48241 | Meth sus pneum d/t Staph | Methicillin susceptible pneumonia due to Staphylococcus aureus |
| 48242 | Meth res pneu d/t Staph | Methicillin resistant pneumonia due to Staphylococcus aureus |
| 481 | Pneumococcal pneumonia | Pneumococcal pneumonia [Streptococcus pneumoniae pneumonia] |
| 48282 | Pneumonia e coli | Pneumonia due to escherichia coli [E. coli] |
| 4822 | H.influenzae pneumonia | Pneumonia due to Hemophilus influenzae [H. influenzae] |
| 4820 | K. pneumoniae pneumonia | Pneumonia due to Klebsiella pneumoniae |
| 48284 | Legionnaires' disease | Pneumonia due to Legionnaires' disease |
| 48283 | Pneumo oth grm-neg bact | Pneumonia due to other gram-negative bacteria |
| 4821 | Pseudomonal pneumonia | Pneumonia due to Pseudomonas |
| 48240 | Staphylococcal pneu NOS | Pneumonia due to Staphylococcus, unspecified |
| 4846 | Pneum in aspergillosis | Pneumonia in aspergillosis |
| 4841 | Pneum w cytomeg incl dis | Pneumonia in cytomegalic inclusion disease |
| 486 | Pneumonia, organism NOS | Pneumonia, organism unspecified |
| 5070 | Food/vomit pneumonitis | Pneumonitis due to inhalation of food or vomitus |
| 5078 | Solid/liq pneumonit NEC | Pneumonitis due to other solids and liquids |
| 99732 | Postproc aspiration pneu | Postprocedural aspiration pneumonia |
| 99731 | Ventltr assoc pneumonia | Ventilator associated pneumonia |
| ICD-9, international classification of diseases-9. | | |
