# Supplementary material for: Biological signatures and prediction of an immunosuppressive status—persistent critical illness—among orthopedic trauma patients using machine learning techniques
Source: Front Immunol. 2022 Oct 17;13:979877. doi: 10.3389/fimmu.2022.979877 (PMC9620964; doi:10.3389/fimmu.2022.979877)
Supplement: Supplementary file 3 [file Table_3.docx]

| **Supplementary table 3 \|** Patient’s demographic and clinical characteristics in the external validation cohort. | |
| --- | --- |
| **Characteristics** | **Overall** |
| n | 113 |
| Age (median [IQR]) | 55.00 [47.00, 64.00] |
| Gender (female/male, %) | 35/78 (31.0/69.0) |
| Albumin (g/dL, median [IQR]) | 2.98 [2.65, 3.52] |
| Total calcium (mg/dL, median [IQR]) | 8.36 [7.80, 9.24] |
| Sodium (mEq/L, median [IQR]) | 140.20 [137.70, 143.00] |
| RDW hematology (%, median [IQR]) | 14.30 [13.30, 15.50] |
| pH (units, median [IQR]) | 7.39 [7.35, 7.43] |
| Heart rate (BPM, median [IQR]) | 94.00 [81.00, 108.00] |
| Respiratory failure (no/yes, %) | 81/32 (71.7/28.3) |
| Bacteremia (no/yes, %) | 103/10 (91.2/8.8) |
| Pneumonia (no/yes, %) | 62/51 (54.9/45.1) |
| SOFA (median [IQR]) | 4.00 [2.00, 7.00] |
| IRQ, inter-quartile range; RDW, red blood cell distribution width; BPM, beats per minute; SOFA, the Sepsis-related Organ Failure Assessment score. | |
