# Supplementary material for: Biological signatures and prediction of an immunosuppressive status—persistent critical illness—among orthopedic trauma patients using machine learning techniques
Source: Front Immunol. 2022 Oct 17;13:979877. doi: 10.3389/fimmu.2022.979877 (PMC9620964; doi:10.3389/fimmu.2022.979877)
Supplement: Supplementary file 4 [file Table_4.docx]

| **Supplementary table 4 \|** Dynamic change of lymphocytes (%) over time between patients with and without persistent critical illness. | | | |
| --- | --- | --- | --- |
| **Dyas after admission** | **Overall (n=820)** | **Persistent critical illness** | |
|  |  | **No** | **Yes** |
| Day 0 | 9.30 [6.00, 15.80] | 9.25 [6.00, 15.10] | 9.30 [5.60, 16.90] |
| Day 1 | 8.30 [4.05, 14.78] | 9.00 [5.07, 14.53] | 4.20 [4.00, 16.15] |
| Day 2 | 11.95 [5.68, 16.90] | 11.55 [5.55, 15.72] | 13.00 [7.38, 23.00] |
| Day 3 | 9.15 [5.00, 17.92] | 9.80 [5.00, 18.50] | 5.45 [5.30, 8.95] |
| Day 4 | 9.50 [6.80, 15.50] | 15.35 [9.05, 19.55] | 6.80 [6.00, 8.00] |
| Day 5 | 10.75 [8.30, 18.82] | 11.10 [7.80, 22.55] | 9.80 [9.50, 17.45] |
| Day 6 | 12.00 [7.65, 15.30] | 10.95 [6.25, 18.73] | 13.00 [11.00, 13.00] |
| Day 7 | 9.55 [6.60, 17.40] | 10.75 [6.40, 18.52] | 7.30 [7.30, 17.40] |
| Day 8 | 9.20 [6.60, 12.00] | 11.00 [7.90, 12.90] | 8.60 [5.95, 12.00] |
| Day 9 | 11.00 [5.75, 19.35] | 11.75 [5.47, 21.35] | 11.00 [6.00, 14.30] |
| Day 10 | 10.50 [6.00, 13.50] | 6.65 [4.82, 17.50] | 10.50 [10.50, 10.60] |
| Day 11 | 12.20 [7.80, 20.75] | 19.00 [9.90, 21.50] | 10.45 [6.60, 16.50] |
| Day 12 | 14.00 [6.73, 23.00] | 23.00 [6.55, 23.00] | 9.90 [7.10, 14.30] |
| Day 13 | 7.90 [3.80, 12.40] | 7.00 [5.00, 17.80] | 9.25 [1.90, 12.40] |
| Day 14 | 16.00 [5.00, 26.00] | 26.00 [15.50, 27.20] | 3.00 [3.00, 5.00] |
| Day 15 | 8.10 [5.40, 15.00] | 8.10 [6.50, 19.80] | 8.65 [5.00, 15.00] |
| Day 16 | 14.00 [13.70, 20.30] | 14.00 [13.70, 26.12] | 6.00 [6.00, 14.00] |
| Day 17 | 15.20 [12.45, 16.20] | 16.20 [15.52, 48.65] | 14.00 [9.50, 14.00] |
| Day 18 | 12.00 [6.25, 18.58] | 71.20 [33.17, 71.20] | 12.00 [5.25, 12.00] |
| Day 19 | 14.00 [9.60, 36.80] | 14.00 [8.50, 75.00] | 15.10 [9.60, 21.80] |
| Day 20 and above | 16.40 [8.97, 22.33] | 20.70 [12.60, 24.67] | 11.00 [7.53, 17.30] |
| Data were presented by median and inter-quartile range. | | | |
