# Supplementary material for: Biological signatures and prediction of an immunosuppressive status—persistent critical illness—among orthopedic trauma patients using machine learning techniques
Source: Front Immunol. 2022 Oct 17;13:979877. doi: 10.3389/fimmu.2022.979877 (PMC9620964; doi:10.3389/fimmu.2022.979877)
Supplement: Supplementary file 5 [file Table_5.docx]

| **Supplementary table 5 \|** Comparison of clinical characteristics between patients with and without persistent critical illness in the external validation cohort. | | | |
| --- | --- | --- | --- |
| **Characteristics** | **Persistent critical illness** | | **P** |
|  | **No** | **Yes** |  |
| n | 82 | 31 |  |
| Age (median [IQR]) | 54.50 [46.00, 70.75] | 55.00 [47.50, 62.00] | 0.740 |
| Gender (female/male, %) | 28/54 (34.1/65.9) | 7/24 (22.6/77.4) | 0.338 |
| Albumin (g/dL, median [IQR]) | 3.15 [2.75, 3.59] | 2.71 [2.54, 3.04] | 0.002 |
| Total calcium (mg/dL, median [IQR]) | 8.50 [7.82, 9.53] | 8.04 [7.72, 8.38] | 0.029 |
| Sodium (mEq/L, median [IQR]) | 140.30 [138.00, 143.00] | 140.10 [136.85, 143.00] | 0.895 |
| RDW hematology (%, median [IQR]) | 14.40 [13.33, 15.90] | 14.20 [13.25, 15.05] | 0.442 |
| pH (units, median [IQR]) | 7.40 [7.36, 7.44] | 7.34 [7.30, 7.40] | <0.001 |
| Heart rate (BPM, median [IQR]) | 90.00 [78.25, 107.75] | 104.00 [93.00, 113.50] | 0.003 |
| Respiratory failure (no/yes, %) | 67/15 (81.7/18.3) | 14/17 (45.2/54.8) | <0.001 |
| Bacteremia (no/yes, %) | 78/4 (95.1/4.9) | 25/6 (80.6/19.4) | 0.041 |
| Pneumonia (no/yes, %) | 55/27 (67.1/32.9) | 7/24 (22.6/77.4) | <0.001 |
| SOFA (median [IQR]) | 4.00 [2.00, 5.75] | 6.00 [4.50, 8.50] | 0.001 |
| IRQ, inter-quartile range; RDW, red blood cell distribution width; BPM, beats per minute; SOFA, the Sepsis-related Organ Failure Assessment score. | | | |
