# Supplementary material for: Biological signatures and prediction of an immunosuppressive status—persistent critical illness—among orthopedic trauma patients using machine learning techniques
Source: Front Immunol. 2022 Oct 17;13:979877. doi: 10.3389/fimmu.2022.979877 (PMC9620964; doi:10.3389/fimmu.2022.979877)
Supplement: Supplementary file 6 [file Table_6.docx]

| **Supplementary table 6 \|** Machine learning algorithms and full super-parameters. | |
| --- | --- |
| **Algorithms** | **Parameters** |
| Gradient Boosting Classifier | GradientBoostingClassifier(max_depth=48, max_features='log2', min_samples_leaf=132, min_samples_split=149, n_estimators=103, random_state=42) |
| Random Forest Classifier | RandomForestClassifier(max_depth=51, max_features='auto', min_samples_leaf=22, min_samples_split=74, n_estimators=39, random_state=42) |
| Decision Tree Classifier | DecisionTreeClassifier(max_depth=6, max_features='log2', min_samples_leaf=29, min_samples_split=109, random_state=42) |
| Support Vector Machine Classifier | SVC(C=1, kernel='poly', probability=True, random_state=42) |
| Logistic Regression Classifier | LogisticRegression(C=10, random_state=42) |
