# Supplementary figures and images for: Biological signatures and prediction of an immunosuppressive status—persistent critical illness—among orthopedic trauma patients using machine learning techniques
Source: Front Immunol. 2022 Oct 17;13:979877. doi: 10.3389/fimmu.2022.979877 (PMC9620964; doi:10.3389/fimmu.2022.979877)

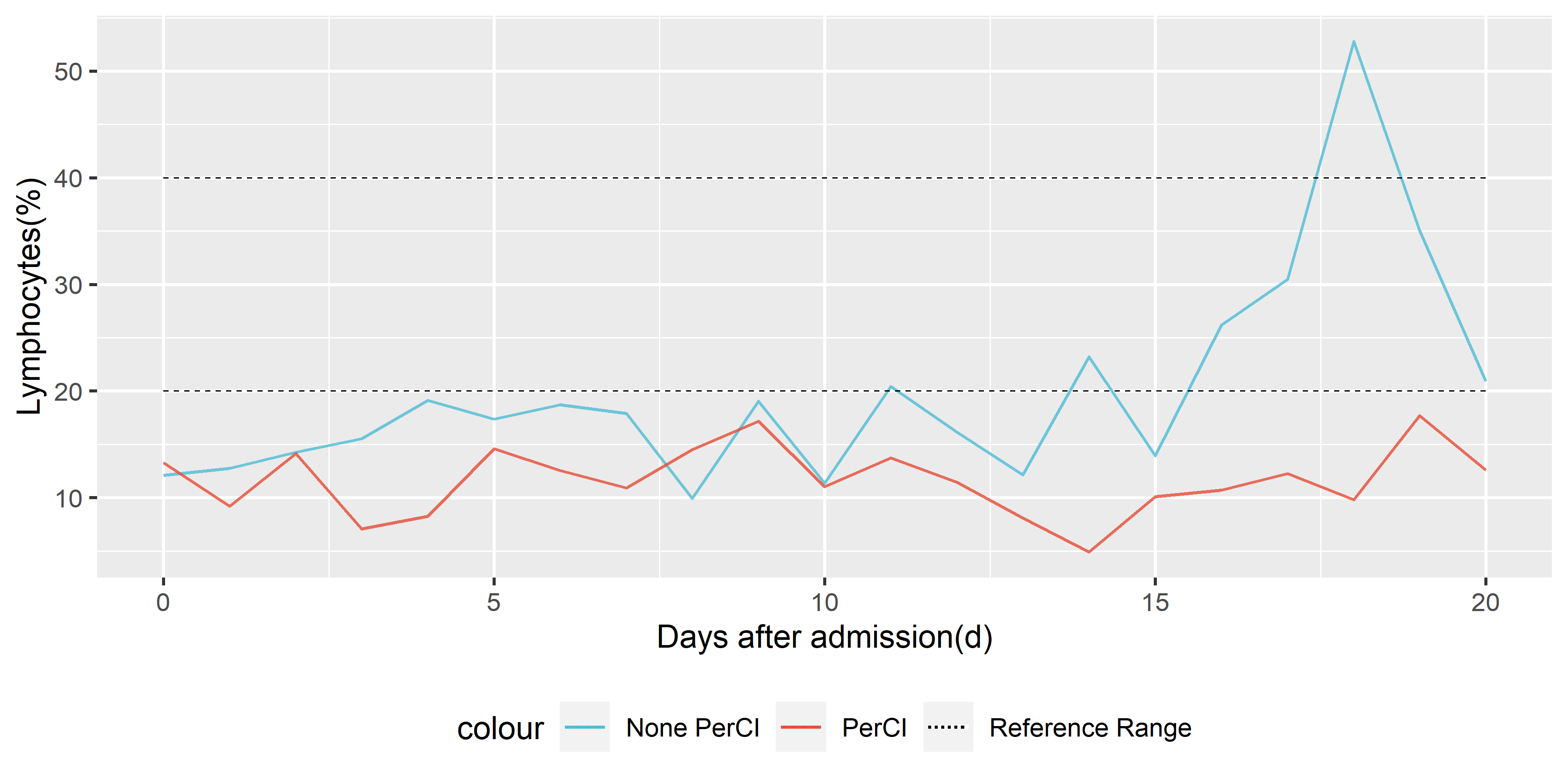

Supplement: Supplementary Figure 1 — Line chart for the dynamic changes of the mean level of lymphocytes (%) with time after ICU admission between patients with (red line) and without (sky-blue line) persistent critical illness. The black dotted line indicates normal reference range. [file Image_1.tif]

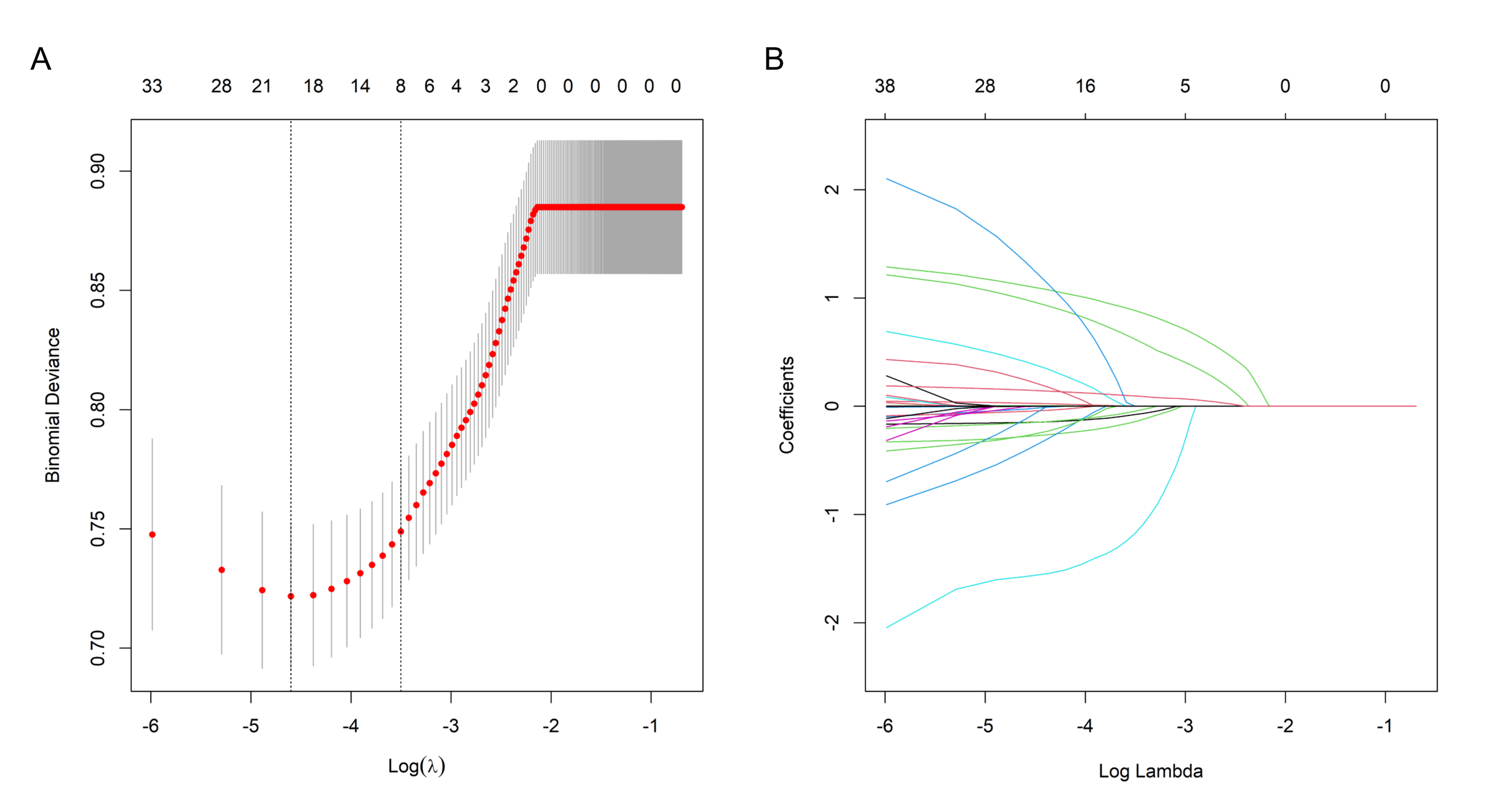

Supplement: Supplementary Figure 2 — Selection of model predictors using the least absolute shrinkage and selection operator (LASSO) logistic regression model combined with 10-fold cross-validation. (A) Selection of appropriate parameters (λ) from the LASSO method by ten-fold cross-validation and minimum criteria. Binomial deviance curve was plotted against log (λ). Dotted vertical lines were drawn at the optimal values by the minimum criteria and the one standard error of the minimum criteria; (B) LASSO coefficient profiles of all variables. Coefficient curve was plotted against the log lambda sequence. A vertical line was presented at the value. [file Image_2.tif]

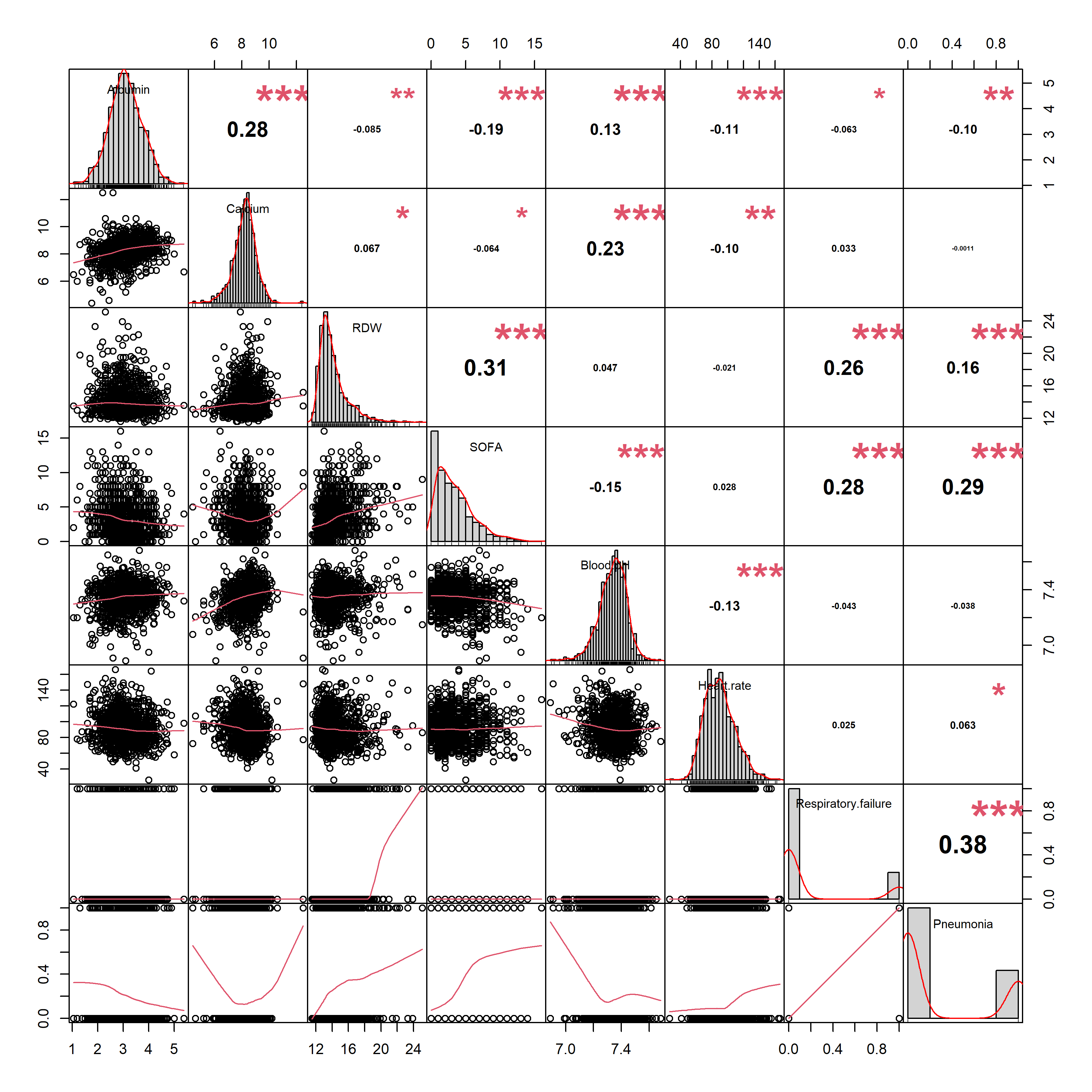

Supplement: Supplementary Figure 3 — Correlation matrix for model predictors. Panels in the lower left show scatted diagrams between the eight model predictors, and smooth lines (red) were fitted. Panels in the upper right show correlation coefficients between the eight predictors. Panels in the diagonal line show histograms of each predictor. * indicates P<0.05, ** indicates P<0.01, *** indicates P<0.001. [file Image_3.tif]

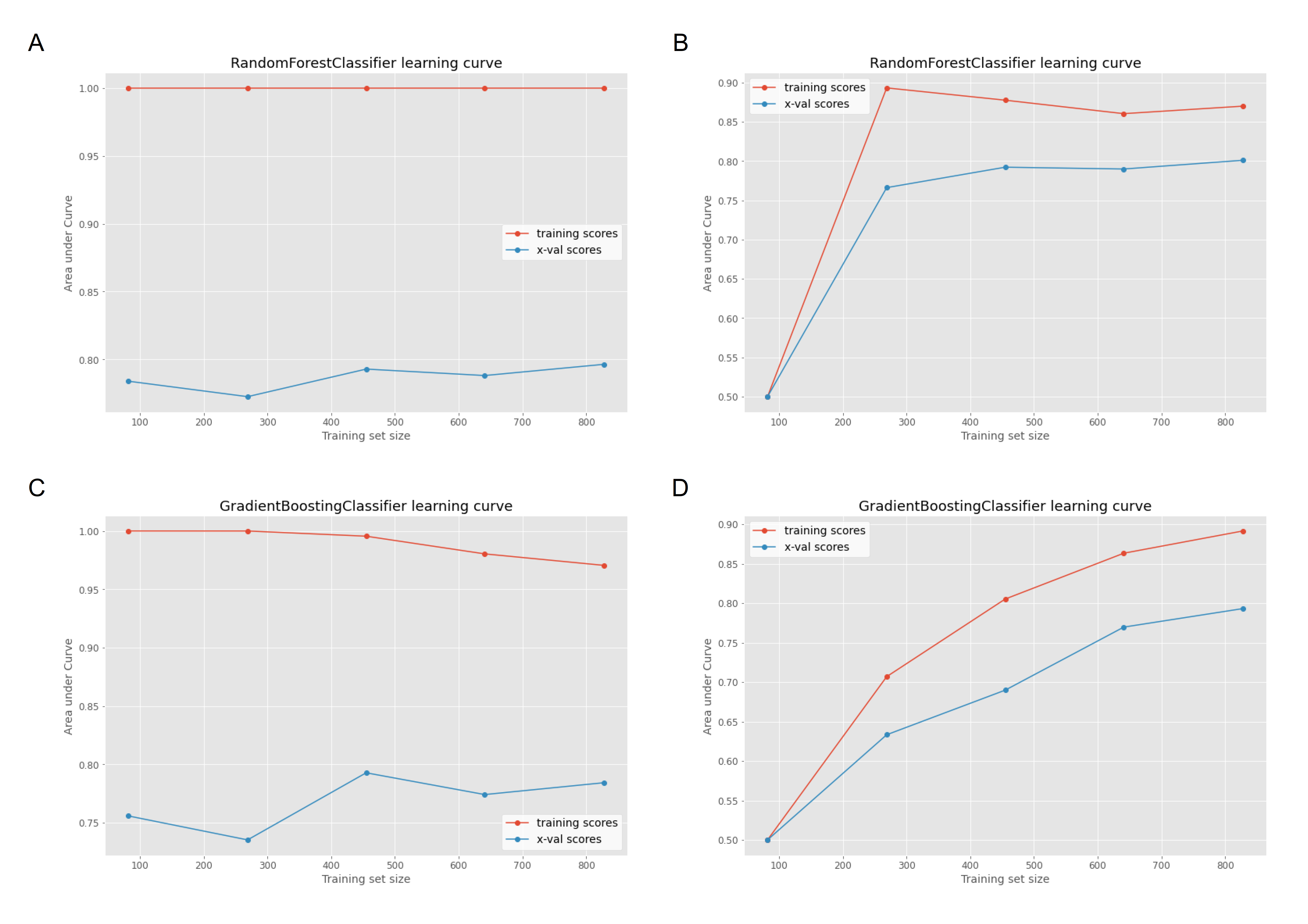

Supplement: Supplementary Figure 4 — Learning curves for machine learning. (A) Random Forest; (B) Gradient Boosting Machine. [file Image_4.tif]

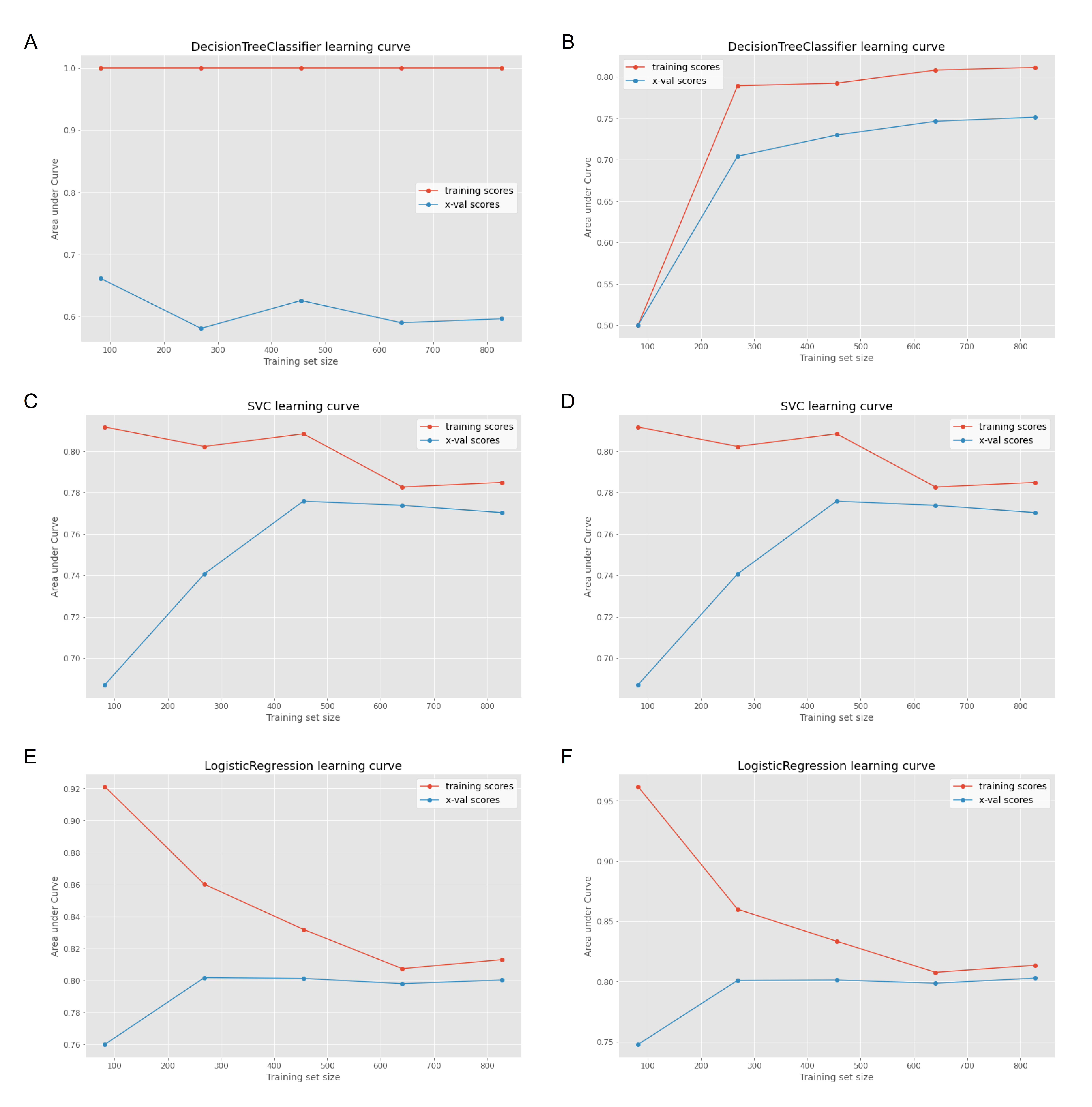

Supplement: Supplementary Figure 5 — Learning curves for machine learning. (A) Decision Tree; (B) Support Vector Machine; (C) Logistic Regression. [file Image_5.tif]

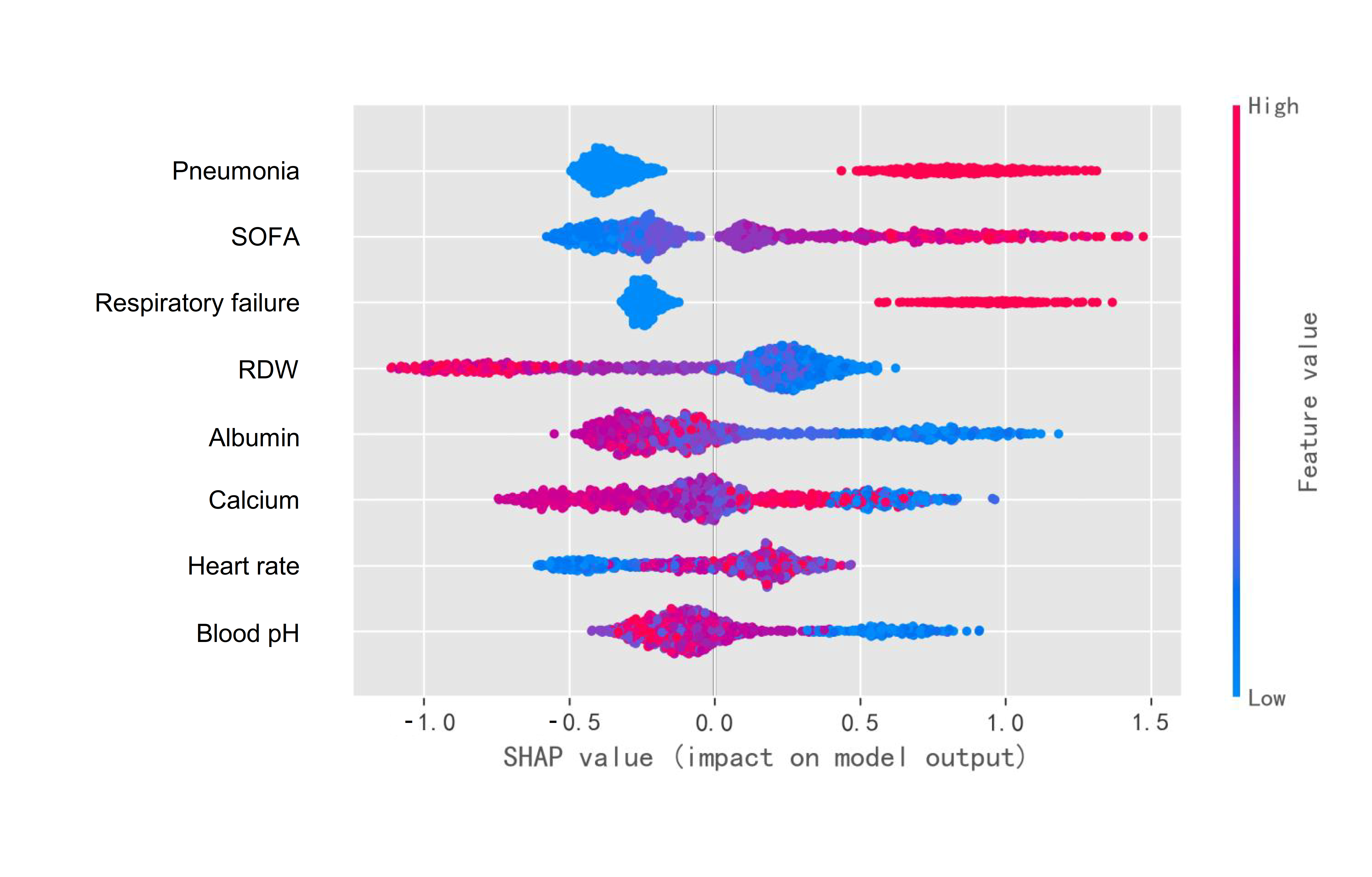

Supplement: Supplementary Figure 6 — Analysis of model predictors in terms of SHapley Additive exPlanations. It presents how the Shapley value distribute in each predictor. The color represents the eigenvalue, and red indicates high value (risk contributor to the outcome) and blue indicates low value (protective contributor to the outcome) in the study. [file Image_6.tif]

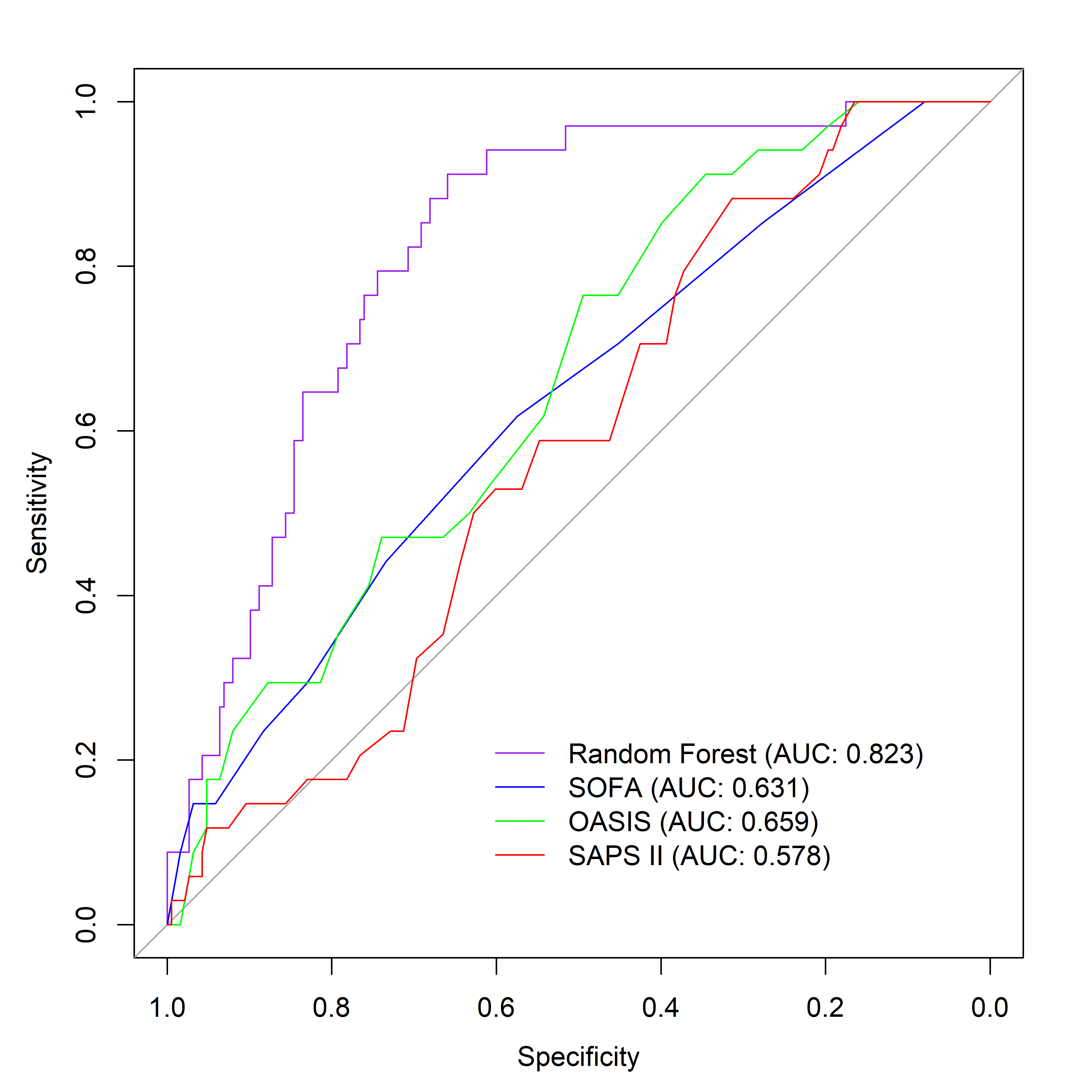

Supplement: Supplementary Figure 7 — Area under the receiver operating characteristic curve (AUROC) for a comparison among the random forest algorithm, the Oxford Acute Severity of Illness Score (OASIS), the Simplified Acute Physiology Score II (SAPS-II), and the Sepsis-related Organ Failure Assessment score (SOFA). [file Image_7.tif]

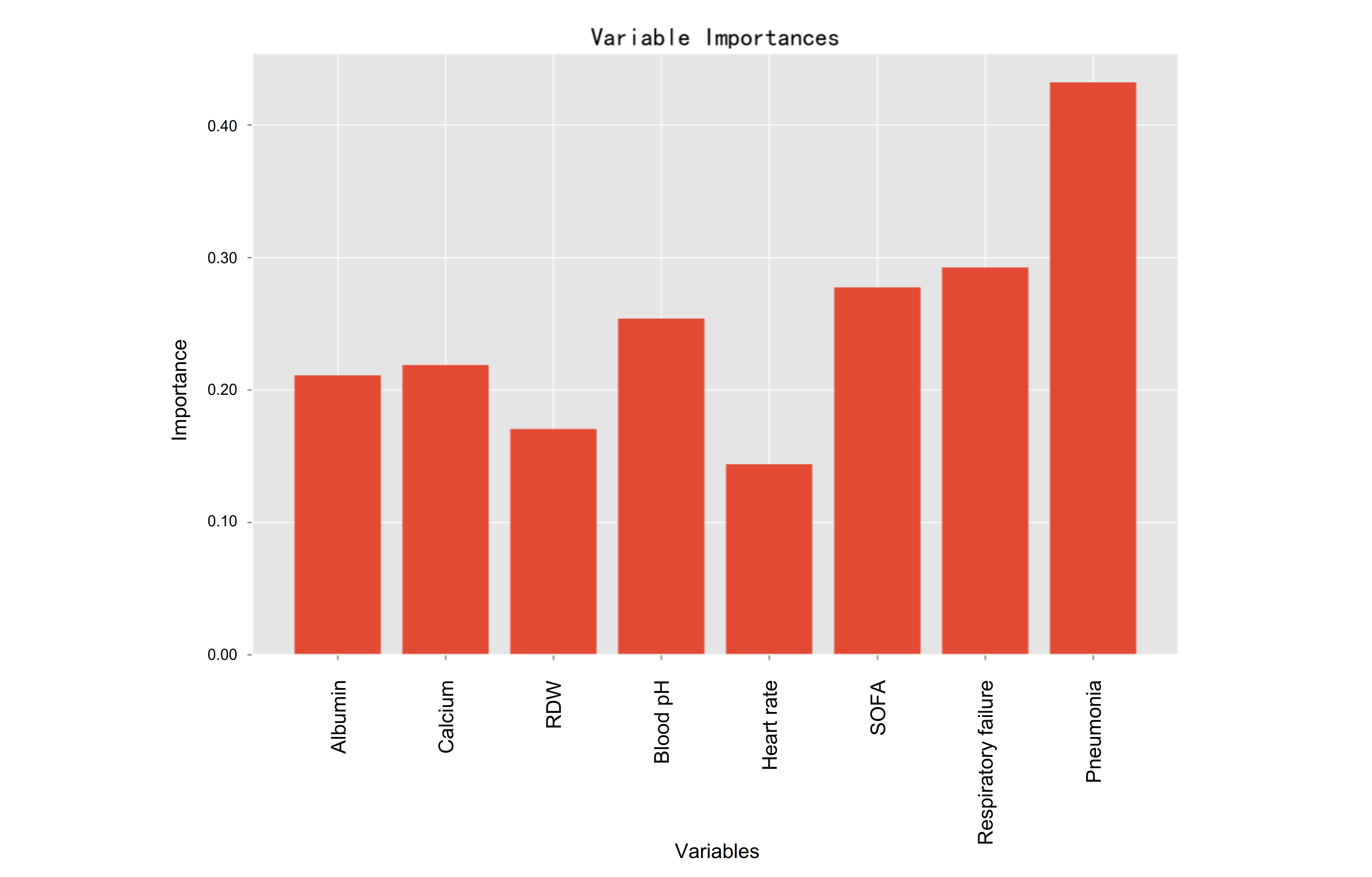

Supplement: Supplementary Figure 8 — Feature importance of model predictors in terms of the gradient boosting machine algorithm. [file Image_8.tif]

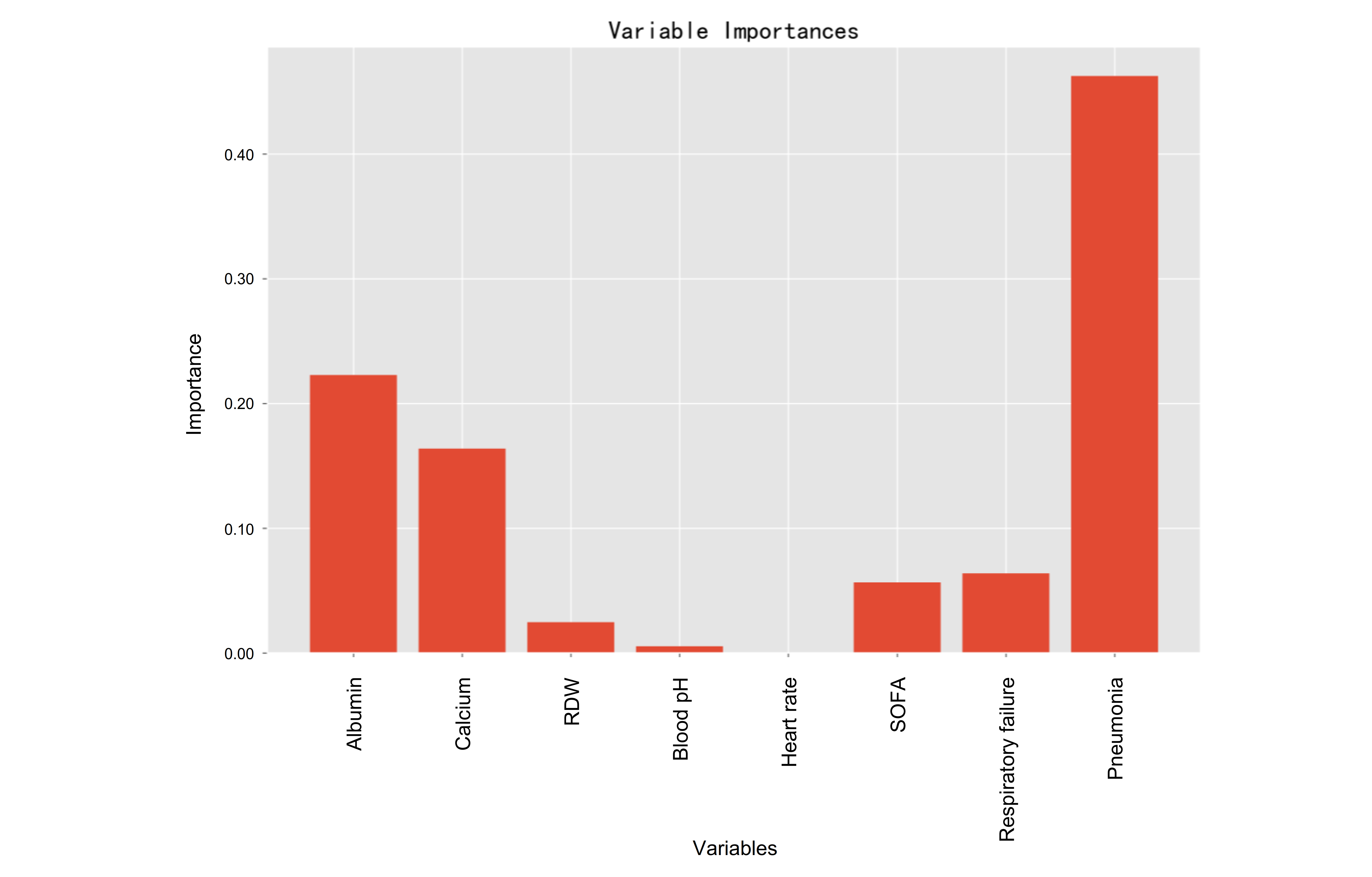

Supplement: Supplementary Figure 9 — Feature importance of model predictors in terms of the decision tree algorithm. [file Image_9.tif]

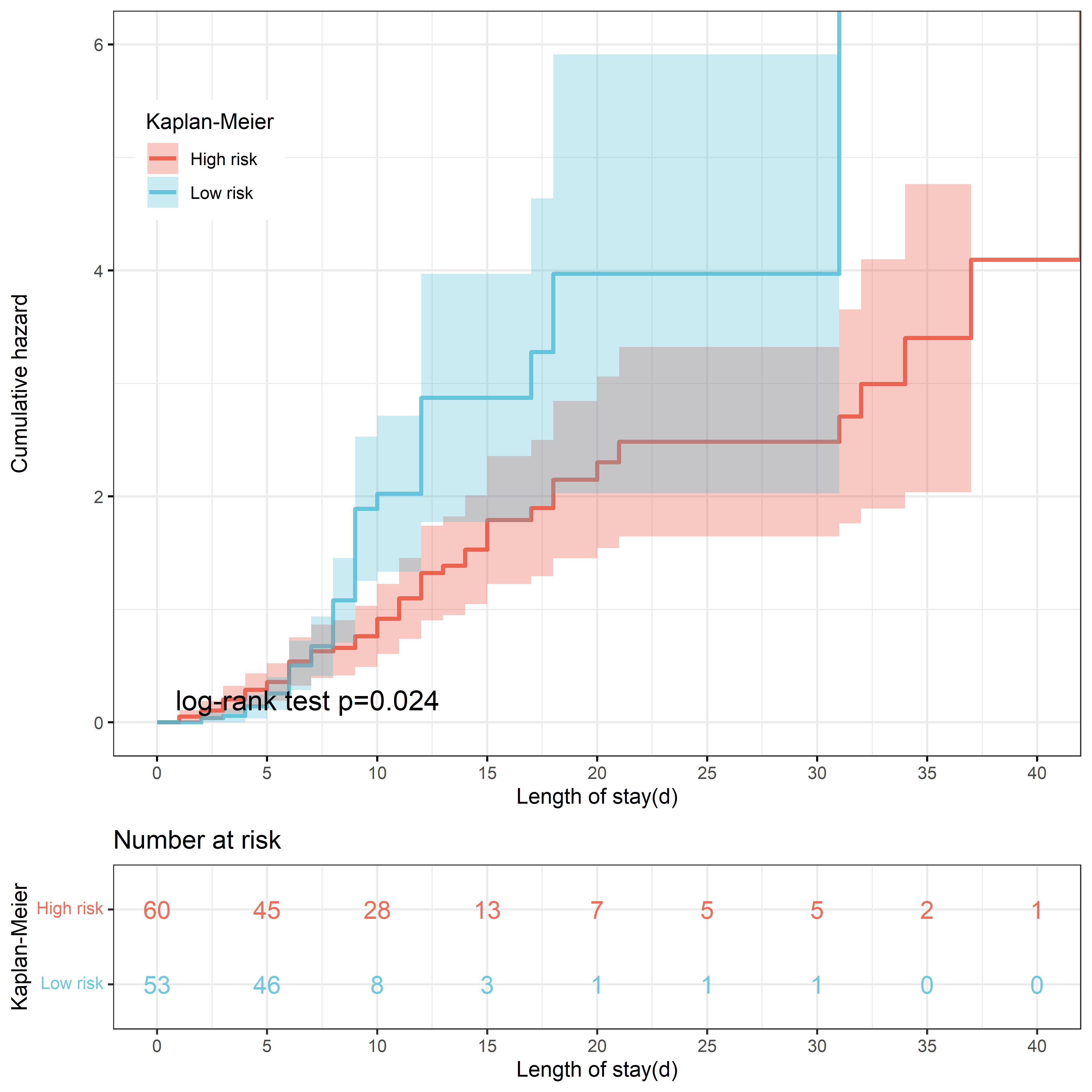

Supplement: Supplementary Figure 10 — Kaplan-Meier curve shows cumulative hazard stratified by risk stratification system in the external validation cohort (P=0.024, Log-rank test). “Sky bule” indicates patients in the low-risk group; “Red” indicates patients in the high-risk group. [file Image_10.tif]

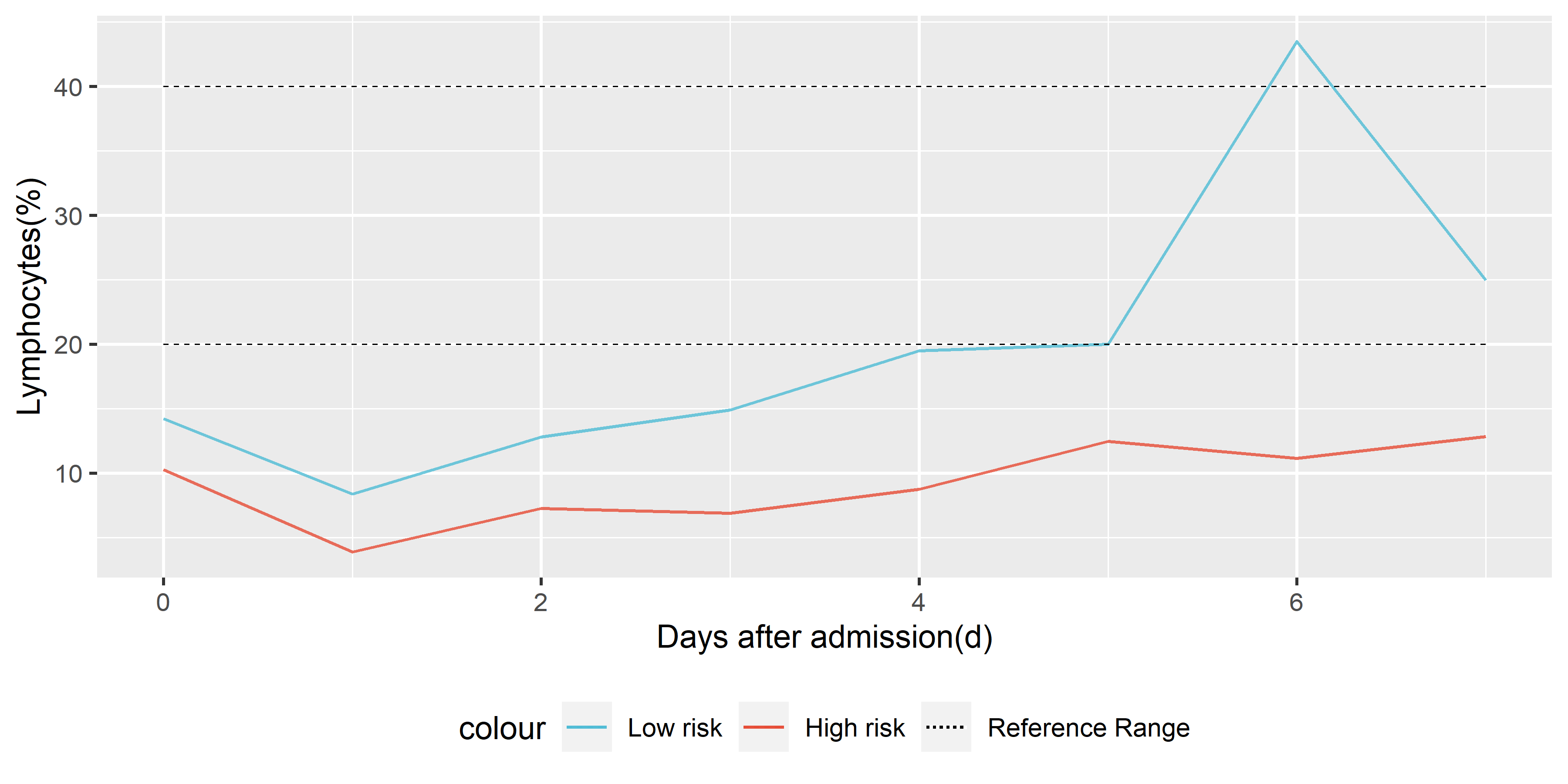

Supplement: Supplementary Figure 11 — Line chart for the dynamic changes of the mean level of lymphocytes (%) with time after ICU admission between patients in the low-risk group (sky-blue line) and patients in the high-risk group (red line). The black dotted line indicates normal reference range. [file Image_11.tif]

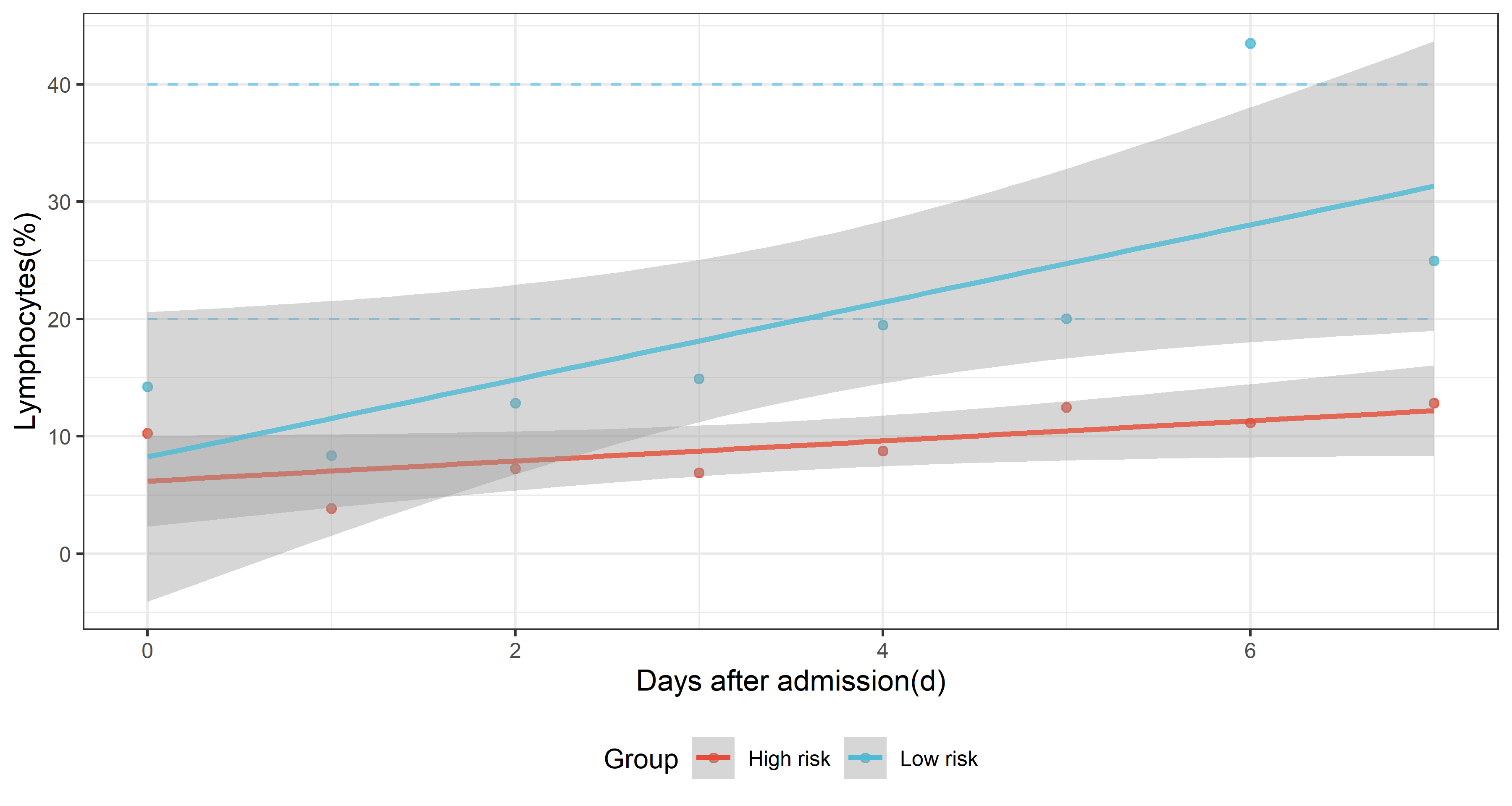

Supplement: Supplementary Figure 12 — Dynamic changes of the mean level of lymphocytes (%) with time after ICU admission between patients in the low-risk group (sky-blue dots) and patients in the high-risk group (red dots). The blue-sky dotted line indicates normal reference range. The red and sky-blue dots are fitted by smooth lines respectively. [file Image_12.tif]
